# Investigating Population Genetic Structure in a Highly Mobile Marine Organism: The Minke Whale *Balaenoptera acutorostrata acutorostrata* in the North East Atlantic

**DOI:** 10.1371/journal.pone.0108640

**Published:** 2014-09-30

**Authors:** María Quintela, Hans J. Skaug, Nils Øien, Tore Haug, Bjørghild B. Seliussen, Hiroko K. Solvang, Christophe Pampoulie, Naohisa Kanda, Luis A. Pastene, Kevin A. Glover

**Affiliations:** 1 Dept. of Population Genetics, Institute of Marine Research, Bergen, Norway; 2 BIOCOST Research Group, Dept. of Animal Biology, Plant Biology and Ecology, University of A Coruña, A Coruña, Spain; 3 Department of Mathematics, University of Bergen, Bergen, Norway; 4 Dept. of Marine Mammals, Institute of Marine Research, Bergen, Norway; 5 Dept. of Marine Mammals, Institute of Marine Research, Tromsø, Norway; 6 Marine Research Institute of Iceland, Reykjavik, Iceland; 7 Institute of Cetacean Research, Tokyo, Japan; 8 Department of Informatics, Faculty of Mathematics and Natural Sciences, University of Bergen, Bergen, Norway; Institute of Biochemistry and Biology, Germany

## Abstract

Inferring the number of genetically distinct populations and their levels of connectivity is of key importance for the sustainable management and conservation of wildlife. This represents an extra challenge in the marine environment where there are few physical barriers to gene-flow, and populations may overlap in time and space. Several studies have investigated the population genetic structure within the North Atlantic minke whale with contrasting results. In order to address this issue, we analyzed ten microsatellite loci and 331 bp of the mitochondrial D-loop on 2990 whales sampled in the North East Atlantic in the period 2004 and 2007–2011. The primary findings were: (1) No spatial or temporal genetic differentiations were observed for either class of genetic marker. (2) mtDNA identified three distinct mitochondrial lineages without any underlying geographical pattern. (3) Nuclear markers showed evidence of a single panmictic population in the NE Atlantic according STRUCTURE's highest average likelihood found at K = 1. (4) When K = 2 was accepted, based on the Evanno's test, whales were divided into two more or less equally sized groups that showed significant genetic differentiation between them but without any sign of underlying geographic pattern. However, mtDNA for these individuals did not corroborate the differentiation. (5) In order to further evaluate the potential for cryptic structuring, a set of 100 *in silico* generated panmictic populations was examined using the same procedures as above showing genetic differentiation between two artificially divided groups, similar to the aforementioned observations. This demonstrates that clustering methods may spuriously reveal cryptic genetic structure. Based upon these data, we find no evidence to support the existence of spatial or cryptic population genetic structure of minke whales within the NE Atlantic. However, in order to conclusively evaluate population structure within this highly mobile species, more markers will be required.

## Introduction

Anthropogenic activities are key factors affecting wildlife populations, including altering population structure and distribution patterns [1]–[4]. Overexploitation by the whaling industry led to serious declines in many of the world's populations of whales. Currently, the IUCN conservation status “least concern” is applicable to only ∼20% of whale species and only 8% show increasing population trends [5]. Marine mammals are highly mobile and may travel large distances (*e.g*. Stevick *et al*. [6]). A number of factors are thought to play a role in shaping the genetic structuring of cetacean populations such as the complex social structure (*e.g.* matrilineal based groups), the resource specialization and the great capacity for learning [7]–[9].

Minke whales, the second smallest baleen whales (about 10 m in length), are currently considered as two species [10]: the cosmopolitan common minke whale (*Balaenoptera acutorostrata*, Lacepede, 1804) and the Antarctic minke whale (*B. bonaerensis*, Burmeister, 1867), which is confined to the Southern hemisphere with the exceptions of rare inter-oceans migration events [11], [12]. The former is further divided into three sub-species: the North Atlantic (*B.a. acutorostrata*), the North Pacific (*B.a. scammoni*), and the dwarf common minke whale (*B.a.* unnamed sub-species), which is thought to be restricted to the Southern hemisphere.

*B.a. acutorostrata* occurs in the entire North Atlantic during the Northern hemisphere summer months, limited in the northern range by the ice [13]. Although their winter distribution and thus the location of breeding areas is unknown, they probably fit the general ecological pattern of large cetaceans in the Northern hemisphere and migrate to lower latitudes, inhabiting temperate and tropical waters where pairing and birth of calves takes place [14]. Calves are born between November and March after a gestation period of ten months [15], [16]. In the western North Pacific, two *B.a. scammoni* breeding populations on either side of Japan are known to mix on feeding grounds in the Okhotsk Sea [17].

The minke whale is still harvested in significant numbers, and the management of *B.a. acutorostrata* in the North Atlantic is regulated under the Revised Management Procedure (RMP) developed by the Scientific Committee of the International Whaling Commission which also regularly reviews the species status through Implementation Reviews, the last one completed in 2009 [18]. The RMP implements the concept of Management Areas, which are currently outlined by taking into account different factors including distribution, life history parameters, local conservation threats such as bycatch, pollution, direct human exploitation and competition with fisheries, as well as differences in national legislation [19]. Five Management Areas have been established in the Eastern North Atlantic (*i.e.* “IWC Small Areas” [20]). The main concern of this outline is that, for minke whales, which is a migratory species, the small Management Areas would not reflect the real population boundaries but instead temporary mixed assemblages [21]. Therefore, careful assessment of the genetic diversity and genetic structure of the populations is essential to enable any successful conservation strategy.

Distinct breeding populations have not been identified for North Atlantic minke whales. Hence, the assessment of genetic structuring of minke whales within the North Atlantic has been based upon samples collected in the feeding grounds and stranded individuals. The question of population genetic structure within this species remains unresolved with partially conflicting results. There seems to be a general agreement regarding the absence of any clear spatial genetic structuring at mitochondrial level [22]–[25] although the possibility of co-existence of two breeding populations of common minke whales in the North Atlantic was proposed by Palsbøll [26] after finding two main groups of genotypes when analyzing restriction fragment length polymorphism on mtDNA. Likewise, whereas some studies based on nuclear markers [24], [27], [28] failed to reveal any genetic differentiation between individuals from the central and north-eastern parts of the North Atlantic; some other insights based on stable isotopes and heavy metals [29], levels of radioactive caesium ^137^Cs [30], persistent organochlorines [30], microsatellites [23] or isozymes [31]–[33] suggest a geographic substructuring across different areas of the North Atlantic. Recently, Anderwald *et al*. [24], using a set of ten microsatellites, reported the possible existence of two cryptic stocks across the North Atlantic. All these uncertainties regarding population identification and assessment have further increased the scientific and political controversy that whaling already poses [34] and therefore, the need to elucidate population genetic structure within this species.

**Figure 1:**
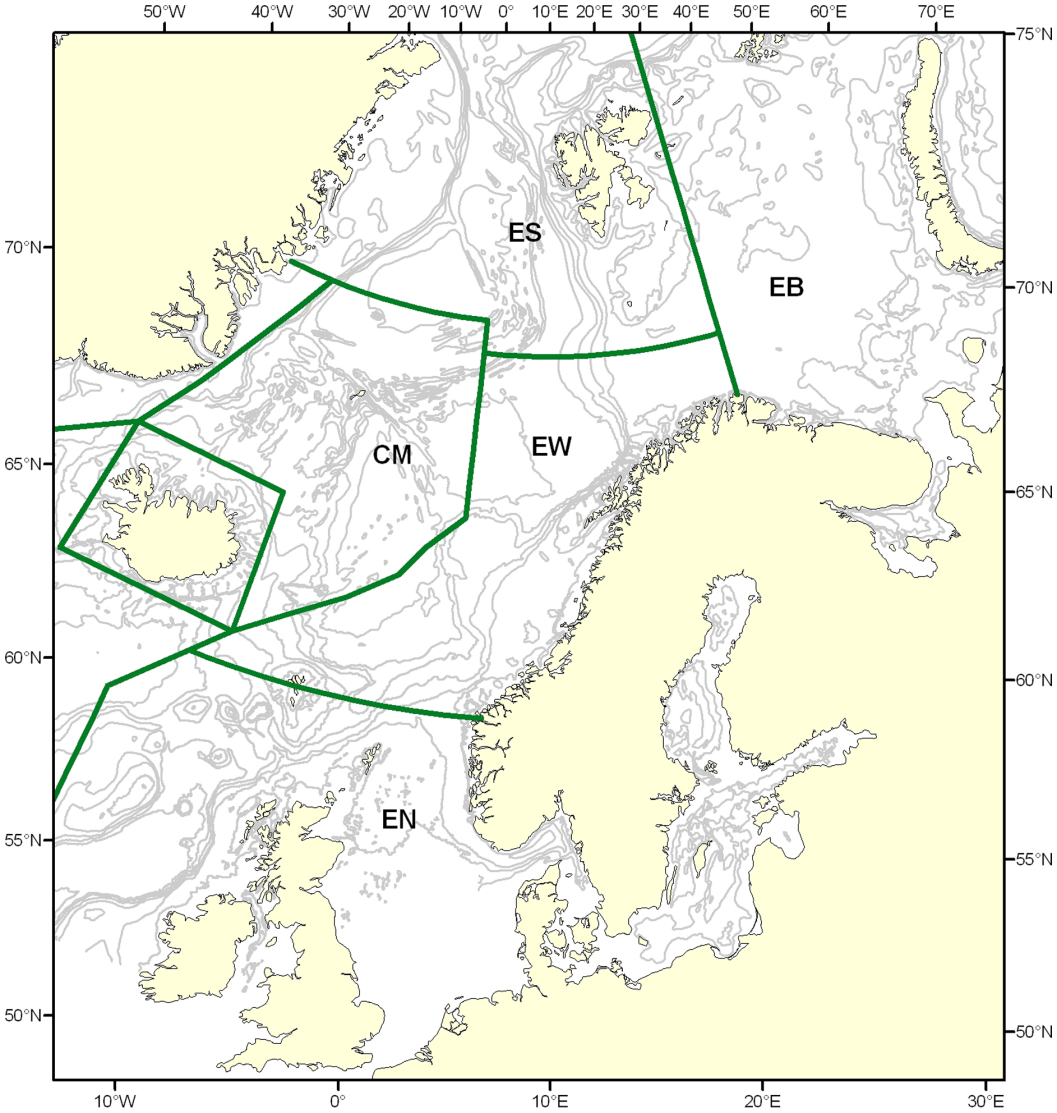
Geographic distribution of the five International Whaling Commission (IWC) Management Areas: ES (Svalbard-Bear Island area), EB (Eastern Barents Sea), EW (Norwegian Sea and coastal zones off North Norway, including the Lofoten area), EN (North Sea), and CM (Western Norwegian Sea-Jan Mayen area).

Norway conducts a commercial harvest of minke whale, *B.a. acutorostrata* in the Northeast Atlantic, and each year, approximately 500 whales are captured across five IWC Management Areas (Fig. 1). In order to enforce domestic regulation and compliance within this harvest, an individual-based DNA register (NMDR) has been maintained since 1996 [12]. This register contains genetic data of ten microsatellites and mtDNA for approximately 8000 whales harvested during the period 1996–2011. In addition, the register includes biometric information together with the geographic position of captures, what provides a powerful database to investigate the potential genetic structure of this species in the NE Atlantic.

The main objective of this study was to investigate spatial and temporal genetic structure of *B. a. acutorostrata* harvested in the NE Atlantic IWC Management Areas during the period 2007–2011. Secondly, we examined the possible existence of cryptic populations distributed across the North Atlantic as proposed by Anderwald *et al*. [24]. Therefore, the present study also included a set of samples from 2004 to match their sampling time frame and hence to enable comparisons. To achieve this second objective, conventional genetic analyses as well as simulation studies were conducted.

## Material and Methods

### Sampling, genotyping, and mtDNA sequencing

The time frame of the present study circumscribes to the period 2007–2011 when the genotyping of the individuals was performed by the Institute of Marine Research in Bergen, following very strict procedures to ensure the data quality [35], [36]. In addition, samples collected in 2004 have been included for comparative purposes with former studies. Thus, the present data consists of genetic data from 2990 whales (2156 females and 834 males) that were harvested in the period from April to September. The distribution of individuals per sex, year and Management Areas is shown in Table 1. No animals were killed to provide samples for the present study as all the samples analyzed existed prior to it and were included in the NMDR [12] from which all the information used in the present work has been obtained; *i.e.* the biometrics, the position of the catches, the microsatellite genotypes and the mtDNA sequences of each of the 2990 individuals that were analyzed. The analytical approaches used for nuclear and mitochondrial markers at the NMDR were the following: DNA was extracted twice from muscle stored in ethanol using Qiagen DNeasy Blood & Tissue Kit following manufacturer's instructions and DNA concentration was measured on a Nanodrop. Ten microsatellite loci: EV1*Pm*, EV037*Mn* [37]; GATA028, GATA098, GATA417 [38]; GT023, GT211, GT310, GT509, GT575 [39] were amplified in three multiplex reactions based on a 2 minute hot start at 94°C, denaturizing for 20 seconds at 94°C, annealing for 45 seconds, elongation at 72°C for 1 minute and a final hold at 4°C. Multiplex specific conditions are detailed in Glover *et al*. [12]. Individuals were sexed using specific primers for the ZFY/ZFX gene [40].

**Table 1:** Distribution of females (F) and males (M) per Management Area (Fig. 1) on a per year class basis.

		MANAGEMENT AREAS										
		EW		ES		EB		EN		CM		
Year	Period	F	M	F	M	F	M	F	M	F	M	Total
2004	25^th^ April – 23^rd^ September	102	83	107	2	100	23	52	29	17	0	515
2007	22^nd^ April – 22^nd^ August	89	83	265	11	8	20	44	47	0	0	567
2008	30^th^ April – 5^th^ September	52	90	212	8	9	11	47	39	25	5	498
2009	11^th^ April - 15^th^ September	84	87	229	14	3	0	24	25	0	0	466
2010	29^th^ April – 11^th^ September	60	80	252	12	11	6	23	4	1	0	449
2011	1^st^ May – 27^th^ August	93	110	160	24	78	18	9	3	0	0	495
	**Total**	**480**	**533**	**1225**	**71**	**209**	**78**	**199**	**147**	**43**	**5**	**2990**

The D-loop region of mtDNA was amplified by performing forward sequencing of one DNA isolate and reverse sequencing of the second one. The first PCR reaction yielded a 1066 bp amplification product, which was forward strand sequenced. The second PCR reaction entailed the amplification of a 331 bp product that was sequenced in the reverse direction. PCR conditions for the two directions were identical, thus containing 0.5 units Go Taq polymerase (Promega), 1.5 mM MgCl_2_, 0.2 mM dNTP and 0.2 µM of each primer. Forward product used primers MT4(M13F) and MT3(M13Rev) modified from Árnason *et al*. [41], whereas primers for the reverse product were: BP15851(M13F), modified from Larsen *et al.* [42] and MN312(M13R), modified from Palsbøll *et al.* [43]. PCR conditions were: hot start at 94°C for 2 min, followed by 30 cycles of denaturizing at 94°C for 50 seconds annealing at 53°C for 50 seconds and elongation at 72°C for 3 min 30 seconds, and finally a 10 min elongation at 72°C and a 4°C hold.

### Genetic structure according to microsatellites

Total number of alleles, allelic richness and the inbreeding coefficient F_IS_ per population and per year were calculated with MSA [44], whereas observed (H_O_) and unbiased expected heterozygosity (UH_E_) were computed with GenAlEx [45]. The genotype distribution of each locus per year class and its direction (heterozygote deficit or excess) was compared with the expected Hardy-Weinberg distribution using the program GENEPOP 7 [46] as was the linkage disequilibrium. Both were examined using the following Markov chain parameters: 10000 steps of dememorisation, 1000 batches and 10000 iterations per batch.

We used several methods to estimate population structure, including STRUCTURE [47], BAPS [48], and traditional F_ST_ [49] and R_ST_ analyses [50]. Slatkin's R_ST_ is an analogue of Wright's F_ST_ [51], adapted to microsatellite loci by assuming a high-rate stepwise mutation model instead of a low-rate K- or infinite-allele mutation model.

Both genetic differentiation among Management Areas per year class, and the level of temporal population genetic differentiation were tested using the Analysis of Molecular Variance (AMOVA) implemented in ARLEQUIN v.3.5.1.2 [52]. We also calculated the pairwise F_ST_ between populations from year class 2004 to 2011.

Both STRUCTURE [47], [53], [54] and BAPS [48], [55] conduct a Bayesian analysis to identify hidden population structure, the former using allele frequency and linkage disequilibrium information from the data set directly, the latter identifying populations with different allele frequencies. Thus, BAPS first infers the most likely individual clusters in the sample population and then performs the most likely admixture of genotypes [55]; an approach that is more powerful in identifying hidden structure within populations [56].

We used the Bayesian model-based clustering algorithms implemented in STRUCTURE v. 2.3.4 to identify genetic clusters under a model assuming admixture and correlated allele frequencies without using population information. Ten runs with a burn-in period consisting of 100000 replications and a run length of 1000000 Markov chain Monte Carlo (MCMC) iterations were performed for a number of clusters ranging from K = 1 to K = 5. If applicable, we then used STRUCTURE Harvester [57] to calculate the Evanno *et al*. [58] *ad hoc* summary statistic ΔK, which is based on the rate of change of the ‘estimated likelihood’ between successive K values. The usual scenario where this approach is appropriate are those cases where once the real K is reached, L(K) at larger Ks plateaus or continues increasing slightly and the variance between runs increases. Hence, the estimated ‘log probability of data’ does not provide a correct estimation of the number of clusters and instead, ΔK accurately detects the uppermost hierarchical level of structure [58]. Runs were automatized with the program ParallelStructure [59] that controls the program STRUCTURE and distributes jobs between parallel processors in order to significantly speed up the analysis time. Afterwards, runs were averaged with CLUMPP version 1.1.1 [60] using the LargeKGreedy algorithm and the G′ pairwise matrix similarity statistics. Averaged runs were graphically displayed using barplots on a per year class basis.

Secondly, we used BAPS 6.0 [48] for a number of clusters ranging between K = 1 and K = 5 (10 runs per K), and then we performed the most likely admixture of genotypes [55], again on a per year class basis.

### Mitochondrial DNA

Estimates of genetic diversity were calculated with DnaSP [61] and consisted of number of segregating sites, average number of pairwise nucleotide differences, nucleotide diversity and haplotype diversity.

Demographic changes were examined using three different approaches: Tajima's D [62], Fu's F_S_ [63] and by comparing mismatch distributions of pairwise nucleotide differences between haplotypes to those expected under a sudden population expansion model [64]–[66]. The analyses were implemented in the program ARLEQUIN v.3.5.1.2, and *P*-values were generated using 10000 simulations.

We used Tajima's D and Fu's Fs to test for shift in the allele frequency spectrum compared to a neutral Wright-Fisher model consistent with population expansion under neutral evolution. The neutrality test Fs [63] has been shown to be a powerful test to detect population growth when large sample sizes are available [67]. Large and negative significant values of Fs indicate an excess of recent mutations (haplotypes at low frequency) compared to those expected for a stable population, which can be interpreted as a signature of recent population growth, genetic hitchhiking or population expansion following a bottleneck event [63]. Demographic changes were also investigated by calculating the raggedness index of the observed mismatch distribution for each of the populations according to the population expansion model. This measure quantifies the smoothness of the observed mismatch distribution. Small raggedness values represent a population which has experienced sudden expansion (possibly following a bottleneck) whereas higher values suggest stationary populations [68], [69]. Unimodal distributions are expected for populations that recently expanded or experienced a bottleneck, as individuals within a population will present similar haplotype divergence (in terms of nucleotide differences) [64], [66]. In contrast, a multimodal or ‘ragged’ distribution is expected for a stable or slowly declining population [64]. Statistical significance for the mismatch distributions was obtained using a goodness-of-fit test based on the sum of squared deviations between the observed and expected distributions [70] and the Harpending's raggedness index, rg [68] after 10000 simulations using the estimated parameters of the expected distribution for a population expansion.

The evolutionary relationships between haplotypes were examined with the software Network [71] using the median-joining algorithm to build an unrooted cladogram. Networks were built separately for every year class and also for the full data set ranging from 2004 to 2011. Singletons were removed, transitions weights were changed into 10 whereas tranversions and gaps were changed into 30; epsilon was set at 10, and the MP option [72] was enabled to delete redundant links and median vectors.

BAPS clustering was used to validate Network results and thus the program was run 100 times for the number of clusters reported by the median-joining tree.

### Testing the hypothesis of cryptic stock clustering of North Atlantic minke whale

Anderwald *et al*. [24] identified genetic sub-structuring of North Atlantic minke whales and proposed the existence of two putative cryptic stocks. In their paper, the detection of genetic differentiation among minke whale individuals was enhanced by the use of an outgroup in STRUCTURE, and thus they included 30 individuals of *B.a. scammoni* from the Sea of Japan as an outgroup. We added this approach to our former STRUCTURE analyses using two different outgroups: firstly, 95 individuals of the subspecies Pacific minke whale (*B. a. scammoni*); secondly, 93 individuals of the Antarctic minke whale (*B. bonaerensis*) and, thirdly, both outgroups simultaneously. STRUCTURE and BAPS analyses together with the assessment of genetic differentiation between groups of individuals after clustering procedures are exhaustively detailed in the File S1 in Supporting Information.

In addition to the above analyses, and to test the alternative hypothesis of minke whales constituting a panmictic population, we created a set of 100 *in silico* generated panmictic populations based on the allele frequencies observed in our samples. Hence, at each of the ten loci, the allelic values (two per individual) were put in a pool, and then randomly re-assigned to individuals, thereby preserving the original allele frequencies. The resulting *in silico* simulated panmictic populations were analysed automatizing STRUCTURE with the program ParallelStructure under a model assuming admixture and correlated allele frequencies without using population information. Ten runs per K ranging from 1 to 5, a burn-in period of 100000 replications, and a run length of 1000000 MCMC iterations were followed by Evanno's test. For the sake of the comparison, ten of the populations yielding K = 2 after Evanno's test were averaged with CLUMPP and pairwise F_ST_ between resulting clusters was performed as above. In addition, BAPS analyses were also conducted for K ranging from 1 to 5.

### Detection of sex-biased dispersal

The potential for sex-biased dispersal was investigated using the microsatellite data with the methods described by Goudet *et al*. [73] and implemented in GenAlEx [45]. The statistics used were: F_IS_, F_ST_, Ho, Hs (the within group gene diversity), the mean corrected assignment index (mAIc) and the variance around the assignment index (vAIc) [74], [75]. When comparing allele frequencies between individuals of the dispersing sex and those of the more philopatric one, a greater similarity is expected among the more dispersing sex. Likewise, expectations would be mAIc to be higher in the more philopatric sex, while vAIc should be lower [73]. Female philopatry and male dispersal are the expected patterns for mammalian species based on the expectation that partuating females will be more dependent on local resources [76]. Thus, a one-tailed Mann–Whitney U-test was used to test if dispersal was biased toward males, as in most marine mammals.

## Results

### Spatial and temporal genetic structure according to microsatellites

The sex distribution per Management Area across years was biased towards females in 73% of the cases (Table 1). Namely, in ES, females were 7–54 fold more abundant than males, whereas in CM no males were reported with the exception of 5 individuals in 2008. The spatial distribution showed that females and males overlapped in latitudes below 71°N but hardly any males occurred in the northernmost regions.

The microsatellite data set contained no missing data and both the number of alleles (116–124), and allelic richness (11.5–12.3) were stable across the six year classes analysed (Table 2). Observed heterozygosity ranged between 0.757 and 0.795, and unbiased expected heterozygosity, between 0.768 and 0.801. Analysis of HWE revealed that at the significance level of α 0.05, 4.8% of loci by sample combinations displayed significant deviations; whereas this number decreased to 0.4% at the significance level of α 0.001. LD was detected 68 times (5.6%) at α 0.05 and 9 (0.7%) at α 0.001.

**Table 2:** Minke whale microsatellites.

Year	No alleles	Ar	No private alleles	Ho	uHe	F_IS_
2004	119	11.7	2	0.757±0.015	0.768±0.010	0.0057±0.0243
2007	120	11.7	0	0.776±0.012	0.770±0.010	−0.0050±0.0185
2008	123	12.1	3	0.791±0.012	0.777±0.010	−0.0150±0.0205
2009	124	12.3	4	0.790±0.018	0.775±0.013	−0.0057±0.0183
2010	116	11.6	0	0.787±0.024	0.801±0.022	0.0093±0.0229
2011	116	11.5	1	0.795±0.017	0.778±0.010	−0.0057±0.0151

Summary statistics per year showing total number of alleles, allelic richness (based on minimum sample size of 449 diploid individuals), number of private alleles, observed heterozygosity (average ± SE), unbiased expected heterozygosity (average ± SE), and inbreeding coefficient (F_IS_) (average ± SD).

The analysis of geographic genetic structuring among Management Areas revealed no differentiation over time. Thus, AMOVA performed separately for each of the year classes reported no significant F_ST_ in any case (Table 3). Likewise, all the pairwise comparisons between Management Areas were non-significant for all year classes analysed with the only exception of EB-EW in 2010 being marginally significant at R_ST_ = 0.024 (*P* = 0.046) but not after Bonferroni correction (*P* = 0.0017).

**Table 3:** Genetic differentiation into Management Areas per year class.

	Microsatellites		mtDNA	
Year	F_ST_	R_ST_	F_ST_ (Haplotype frequency)	F_ST_ (Tamura-Nei)
2004	0.0000 (0.6836)	0.0000 (0.6827)	0.0012 (0.2524)	0.0010 (0.4364)
2007	0.0000 (0.6964)	0.0000 (0.8940)	0.0000 (0.6157)	0.0000 (0.7972)
2008	0.0000 (0.9613)	0.0000 (0.9442)	0.0000 (0.8310)	0.0000 (0.5750)
2009	0.0004 (0.2406)	0.0000 (0.7125)	0.0000 (0.5240)	0.0006 (0.3684)
2010	0.0000 (0.9374)	0.0000 (0.4030)	0.0000 (0.4723)	N.C.NOTE
2011	0.0000 (0.9867)	0.0000 (0.6003)	0.0012 (0.2652)	0.0000 (0.4919)

Summary of AMOVA (F_ST_ and *P*-value) conducted with ARLEQUIN with 10000 permutations at microsatellites and mtDNA.

NOTE N.C. not calculated. Nucleotide composition too unbalanced for Tamura-Nei correction.

Similar to results of spatial genetic structure above, AMOVA reported high temporal genetic stability, and the pairwise comparison between the different year classes (Table 4) yielded only one significant albeit very weak pairwise F_ST_ between years 2007–2008 (F_ST_ = 0.0004, *P* = 0.0270); which was no longer significant after Bonferroni correction.

**Table 4:** Temporal genetic differentiation: Pairwise F_ST_ between year classes calculated with ARLEQUIN for microsatellites (lower diagonal) and mtDNA (upper diagonal).

	2004	2007	2008	2009	2010	2011
**2004**		0.0005	0.0012	0.0005	0.00000	0.0037
**2007**	0.00000		0.0002	0.00000	0.00000	0.0024
**2008**	0.00023	0.00043*		0.00000	0.00000	0.00000
**2009**	0.00000	0.00000	0.00016		0.00000	0.0005
**2010**	0.00000	0.00000	0.00015	0.00000		0.0019*
**2011**	0.00006	0.00000	0.00000	0.00000	0.00018	

Significance calculated after 10000 permutations. Values highlighted in boldface type as significant at P<0.05 (*) lost significance after Bonferroni correction.

The individual analysis of every Management Area also showed temporal genetic stability as a general picture. Hence, the AMOVA performed separately in each of the five IWC zones yielded a non-significant F_ST_ that exhibited 0.003 as the highest value. Likewise, the pairwise comparisons between years within each area were also non-significant with the exception of area EN. The pairwise matrix for EN reached significance in three out of the total fifteen cases corresponding to the comparisons between year 2008 and years 2004, 2007 and 2009 respectively.

STRUCTURE showed that the highest average likelihood was found to be K = 1 in all year classes together with a decreasing trend of LnP(D) across consecutive values of K (Table A in File S1). In these situations, although there is no need to perform Evanno's test; we found that ΔK took its highest value for K = 2 in all the year classes with the exception of 2008 where K = 3. The common feature to all the sampling years were the low values reported for ΔK, which reached its maximum in 2011 (ΔK = 16.7) but otherwise ranged between 3 and 8. Barplots for each year class are shown in Fig. A in File S1. For K≥2, every individual showed that the membership to each cluster was evenly distributed among groups producing flag-like barplots.

BAPS showed that most likely K was 3 for year classes 2008, 2010 and 2011 and 4 for the remaining ones. No admixture was detected in any of the sampling years but in 2004 with one admixed individual.

### Mitochondrial DNA

A total of 92 haplotypes were found in the complete dataset (*i.e.* year classes 2004 and 2007–2011), 25 of them unique (0.8% of the individuals). Six of the haplotypes were shared by 4–9% of the individuals whereas the most abundant one was present in 806 whales (27%). The number of haplotypes found per year class ranged mostly between 36 and 38 (Table 5), and took its maximum value in 2004 (N_H_ = 62). Both haplotype (H_D_) and nucleotide diversity (π) showed high and stable values across the years (Table 5).

**Table 5:** Minke whale mtDNA.

Year	N	N_H_	N_UH_	S	k	Π×10^2^	H_D_
2004	515	62	29	21	3.071	0.969	0.908±0.008
2007	567	49	22	26	2.863	0.906	0.895±0.008
2008	498	38	14	21	2.854	0.900	0.886±0.010
2009	466	36	11	24	2.824	0.891	0.884±0.010
2010	449	38	14	18	2.940	0.931	0.897±0.009
2011	495	37	10	20	2.781	0.877	0.864±0.011

Summary of diversity statistics: Sample size (N), number of haplotypes (N_H_), number of unique haplotypes (N_UH_), number of segregating sites (S), average number of pairwise nucleotide differences (k), nucleotide diversity (π) and haplotype diversity (H_D_, mean ± SD).

The distribution of the most common haplotypes was even across Management Areas and AMOVA revealed that no differentiation was observed among them in any of the sampling years (Table 3), with the exception of the significant pairwise comparison EB-ES (F_ST_ = 0.008, *P* = 0.035) in 2004. Similarly, high temporal stability (Table 4) was reported with one weak but marginally significant pairwise comparison: 2010–2011 (F_ST_ = 0.0019, *P* = 0.043), although not significant after Bonferroni correction.

**Figure 2:**
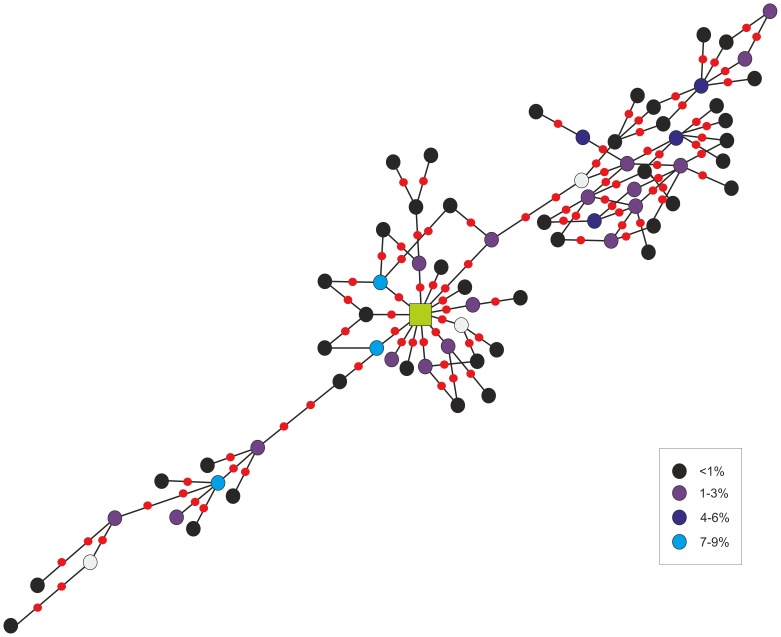
Median-joining network of mtDNA haplotypes corresponding to the period 2004 and 2007–2011. Haplotypes are represented as circles which area is not proportional to its relative frequency for simplicity. Instead, the frequency of haplotypes is depicted through the color code detailed in the legend. The green square represents the ancestral and more abundant haplotype (present in 27% of the individuals). The minimum number of steps connecting parsimoniously two haplotypes is indicated as a red dot, and the open circles represent extinct or missing haplotype that might have not been sampled (mv).

A median-joining tree (Fig. 2) was built for the total data set (*i.e.* 2004 and 2007–2011 excluding singletons) given the temporal stability detected across year classes. A central ancestral haplotype was reported in 27% of the individuals, whereas none of the remaining ones exceeded a frequency of 9%. This ancestral haplotype did not show any phylogeographic structure, *i.e.* it was not linked to any of the Management Areas as it was present in relatively even proportions in each of them. The MJ-tree suggests the existence of three lineages: a central one that evolved through two episodes of expansion, with haplotypes connected between them via single mutational steps in the vast majority of cases. Two of the lineages gathered 85% of the haplotypes in a quite even distribution whereas the third one accounted for the 15% remaining. The haplotype composition per lineages revealed by Network perfectly matched BAPS clustering for K = 3, with 100% coincidence. The same individuals that showed significant differentiation in three lineages at mtDNA yielded F_ST_ = 0.00018 (*P* = 0.8966) when analysed for microsatellites.

Different insights (Table 6) invoke population size expansion such as: a) large negative and significant Fu's Fs, b) negative albeit non-significant Tajima's D values (except in 2010), c) small raggedness values, d) unimodal mismatch distributions (not shown), and e) the star-shape of haplotype network.

**Table 6:** Minke whale mtDNA.

Year	Fu's F_S_	Tajima's D	SSD (*P*-value)	rg (*P*-value)
2004	**−24.916 (0.0002)**	−0.0062 (0.5859)	0.0098 (0.4413)	0.0153 (0.6398)
2007	**−23.861 (0.0000)**	−0.6057 (0.3004)	0.0105 (0.4586)	0.0159 (0.6831)
2008	**−13.279 (0.0119)**	−0.1924 (0.4987)	0.0115 (0.4099)	0.0158 (0.6806)
2009	**−10.710 (0.0279)**	−0.5333 (0.3516)	0.0128 (0.4439)	0.0193 (0.6339)
2010	**−13.711 (0.0104)**	0.2234 (0.6487)	0.0136 (0.3308)	0.0192 (0.5360)
2011	**−12.002 (0.0188)**	−0.1403 (0.5213)	0.0117 (0.4639)	0.0178 (0.6527)

Analyses of population stability (Tajima's D and Fu's F_S_ tests) and population expansion (sum of squared deviations, SSD and raggedness, rg mismatch distribution tests). Significant values are indicated with boldface type.

### Examining potential cryptic population structure using clustering methods

**Figure 3:**
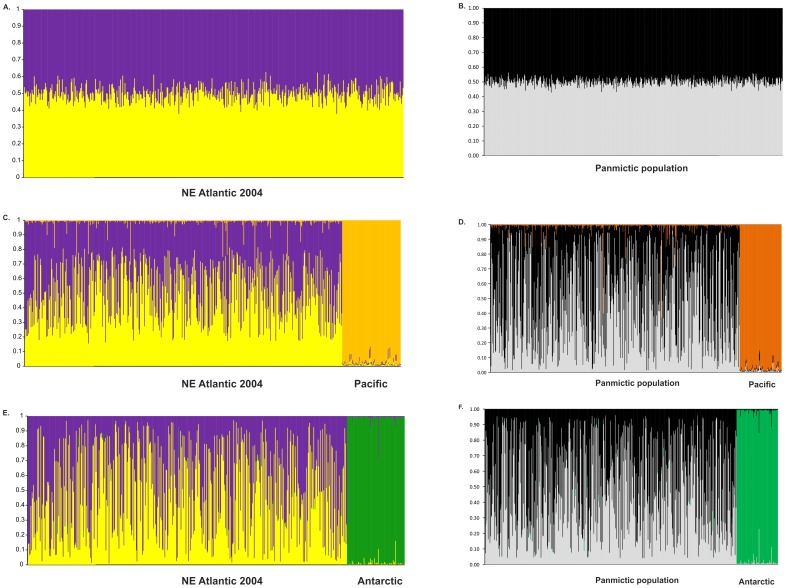
Example of comparison between real populations and the simulated panmictic ones. Bayesian clustering of North East Atlantic minke whale corresponding to year class 2004 (left column) and to a randomly chosen simulated panmictic population (right column). Inferred ancestry of individuals was calculated after averaging ten STRUCTURE runs with CLUMPP for K = 2 (barplots a,b) and K = 3 (barplots c–f). The outgroups were 95 individuals of the Pacific subspecies (*B. a. scammoni*) and 93 individuals of the Antarctic species (*B. bonaerensis*).

Evanno's test revealed that K = 2 was the most likely scenario in all the clustering approaches performed per year class, either with or without outgroups, and even regardless of the number of outgroups included in the analysis (with the exception of year class 2010 without outgroup that showed K = 3). Thus, when using outgroups, NE Atlantic minke whales (*B.a. acutorostrata*) constituted a compact cluster whereas the outgroups (i.e *B.a. scammoni* or/and *B. bonaerensis*) constituted a second compact one (see Fig. B in File S1). Therefore, in such a case, we needed to explore K = 3 to have NE Atlantic minke whales divided into two groups (*e.g*. Fig. 3c–f, and Fig. A3 in File S1). The distribution of those individuals into clusters was conditioned to overcoming a threshold of membership of 0.50. Although an exhaustive report of all the results regarding cryptic clustering can be found in the File S1 in Supporting Information, the main findings of each assignment procedure were as follows:

- **STRUCTURE without outgroup (K = 2).-** Although individuals showed very narrow ranges of membership (0.51–0.63, average 0.59) to clusters (Table B a,b in File S1); there was a significant albeit weak genetic differentiation between groups per year class demonstrated by pairwise F_ST_ (Table B a,b in File S1), Fisher's exact test (*χ* ^2^ =  infinity, df = 20, *P*<0.0001) and Factorial Correspondence Analyses (despite a low percentage of total variation explained by the two first axes ranging between 3.97 and 4.22%). The same individuals genotyped at mtDNA did not produce any significant F_ST_ in any sampling year (Table B a,b in File S1). GeneClass corroborated clustering with an average percentage of correct assignment of 86% (ranging from 83.5 to 90.2%, Table J in File S1).
- **STRUCTURE with outgroups (K = 3).**
  - Pacific minke whale (*B.a. scammoni*) as an outgroup (Fig. C in File S1, left column).- The average membership to cluster was higher than when using STRUCTURE without outgroups (0.88). Individuals were divided into two clusters that showed weak albeit significant genetic differentiation (average F_ST_ between clusters was 0.0130). The same individuals genotyped at mtDNA did not show any genetic differentiation between clusters (Table D in File S1).
  - Antarctic minke whale (*B. bonaerensis*) as an outgroup (Fig. C in File S1, right column).- The average membership to cluster was higher, 0.95, whereas the average F_ST_ between clusters was slightly lower, 0.0122. Again, the individuals from both clusters genotyped at mtDNA did not reveal any genetic differentiation (Table E in File S1).
  - We used a conservative approach and divided the NE Atlantic minke whales into two groups after taking the consensus of the results of the analyses with Antarctic and Pacific outgroups together. This means that individuals were assigned to cluster 1 or 2 after comparing the assignment obtained after Antarctic and Pacific analyses. Likewise, a number of individuals was left unassigned and this comprised those that did not reach the inferred ancestry 0.5 threshold plus the mismatches between both procedures (*e.g.* individuals that belonged to cluster 1 with Antarctic outgroup and to cluster 2 in the Pacific clustering). Again a weak but significant differentiation between groups per year class was shown by pairwise F_ST_ (Table F in File S1), Fisher's exact test (*χ* ^2^ =  infinity, df = 20, *P*<0.0001) and Factorial Correspondence Analyses (albeit the low percentage of the total variation explained by the two first axes ranging from 3.9 to 4.2%). Once more, the same individuals genotyped at mtDNA did not show any evidence of genetic differentiation (Table F in File S1). GeneClass corroborated STRUCTURE consensus clustering with a high percentage of correct assignment (97–98.6%) across all year classes with the exception of 2008 that was slightly lower (85.6%), Table J in File S1.
- **BAPS for K = 2.-** BAPS divided individuals of each sampling year class into two groups of even size for year classes 2008, 2009 and 2011 whereas for the remaining ones, the size ratio was around 1.4–1.5 (Table H in File S1). No admixed individuals were detected in any of the sampling years. A weak albeit significant F_ST_ (Table H in File S1) was found between groups per year class, a differentiation that was further confirmed by Fisher's exact test (*χ* ^2^ =  infinity, df = 20, *P*<0.0001) and Factorial Correspondence Analyses (percentage of the total variation explained by the two first axes ranging between 3.73 and 4.10%). The same individuals genotyped at mtDNA only produced significant F_ST_ for year classes 2007 and 2008 based on Tamura-Nei distance and haplotype frequencies, respectively (Table H in File S1). GeneClass corroborated BAPS clustering with a percentage of correct assignment of 100% in all the cases (Table J in File S1).

The geographic distribution of individuals after both procedures of clustering was slightly different. Hence, STRUCTURE-clustered individuals were evenly distributed among Management Areas per year class whereas for the BAPS-clustered ones, this distribution was less homogeneous in some of the cases (Fig. D and Table K in File S1).

The analyses of the 100 *in silico* generated panmictic populations with STRUCTURE revealed that, again and like the real data: a) the highest average likelihood was detected at K = 1, and b) a decreasing trend of LnP(D) across consecutive values of K was found in all the cases. As formerly reported, even if Evanno's test is not applicable in this situation, we wanted to test its outputs and thus we found that the most likely number of clusters showed different values: K = 2 in 58% of the cases, K = 3 in 33% and K = 4 in the remaining 9%. Similarly, low values of ΔK (ranging from 1 to 15) were also reported for the 100 panmictic populations. CLUMPP was performed on a set of ten randomly chosen simulated populations that showed K = 2 after Evanno's method, and individuals were distributed into clusters after overcoming a threshold of 0.50. In all cases, both clusters showed similar size (ratio 1–1.3) and 7–19% of individuals were left unassigned (Table M in File S1). Although the range of membership to cluster was very low (0.51–0.64), pairwise F_ST_ between groups exhibited low (0.012–0.020) but significant values (*P*<0.0001). Importantly, these values were equal in magnitude to the observations based upon the real data reported above. BAPS analyses showed that, in spite of dealing with panmictic populations, in no case the most likely K was found to be 1. Instead, the number of putative populations took the following values: 3 (4% of the cases), 4 (38%) and 5 (58%) respectively.

### Detection of sex-biased dispersal

According to the expectations that dispersal should be biased towards males, as in most of mammals, mAIc was lower in males than in females (−0.051 *vs*. 0.020) and vAIc was higher (2.90 *vs*. 2.35) Fig. G in File S1, whereas the rest of the statistics (F_IS_, F_ST_, Ho and Hs) took almost identical values in both sexes. However, the Mann–Whitney U-test proved to be non-significant (*P*>0.5) therefore we were unable to detect sex-biased dispersal in North Atlantic minke whales. Furthermore, when performing a two-tailed U-test we found a non-significant result (*P* = 0.953) that would not support a higher female dispersal either.

## Discussion

Overall, the total data set (N = 2990) consisted of 28% males and 72% females; proportions that exactly coincide with Anderwald *et al.* [24] and are very similar to the 21% males 79% females reported by Andersen *et al*. [23]. This uneven presence of sexes was also reflected in the sex composition across Management Areas (Table 1), which also agrees with Andersen *et al*. [23] and Anderwald *et al.* [24] and corresponds to the known segregational behaviour with respect to sex and age during summer as mature females tend to occur further north than males [77]–[79].

Microsatellite loci used here exhibited a range of variation of genetic diversity comparable to what has been formerly reported for the same species [23]–[25] as well as for other balaenopterids such as Bryde's whales, *B. brydei* [80]; fin whales, *B. physalus* [81], [82]; sei whales, *B. borealis* [83]; bowhead whales, *B. mysticetus* [84], [85] and gray whales, *Eschrichtius robustus* [86]. Likewise, a similar magnitude of mtDNA genetic diversity, measured either as nucleotide (average of 0.009) or haplotype diversity (average of 0.9), was formerly reported for *B.a. acutorostrata* within the same geographic area [22]–[25], [43] and resembles what has been described for other whale species [83], [86]–[91]. However, the nucleotide diversity reported for the Antarctic minke whale (*B. bonaerensis*) is higher (0.0159) and this was interpreted as the Antarctic species having larger long-term effective population size than *B.a. acutorostrata* [22]. It has been proposed that the current size of the Antarctic minke whale population is unusually high as an indirect result of the whaling that killed more than 2 million of large whales leading to competitive release for smaller krill-eating species [92].

The mismatch distribution analyses were consistent with exponential population expansion suggesting that populations of North Atlantic minke whale are not at equilibrium, something that had already been reported in the literature for this species in the same geographic area [24], [25]. Earlier studies proposed that this expansion followed the last glacial maximum, as seen for various other cetacean species in the North Atlantic [24].

### Spatial genetic structure

The present molecular markers (ten microsatellite loci and 331 bp of mtDNA D-loop) studied on 2990 individuals congruently failed to reveal any genetic differentiation among Management Areas during the period 2004 and 2007–2011. Only two weak and marginally significant pairwise comparisons were recorded: EB-EW for microsatellites in 2010 (R_ST_ = 0.024, *P* = 0.046) and EB-ES for mtDNA in 2004 (F_ST_ = 0.008, *P* = 0.035). This translates to 1% of pairwise tests showing some spatial and temporal divergence, and neither were significant following Bonferroni correction for multiple testing. This lack of spatial genetic differentiation was the case when analyzing each of the six year classes separately, and also for all the specimens combined in the same AMOVA analysis, both for mitochondrial (F_ST_ = 0.0005, *P* = 0.1134) and nuclear (F_ST_ = 0.0000, *P* = 0.9473) DNA. Likewise, none of the resulting pairwise comparisons between areas were significant. The analyses using the joint data set seems legitimate given the temporal genetic stability found at both markers, stability that had already been reported within similar time frames [23], [25], [27].

The lack of geographic genetic differentiation as revealed in the present study is in agreement with some former studies of minke whales in the N Atlantic that were based on nuclear [24], [25], [27], [28] and mitochondrial DNA markers [22]–[25]. However, the consensus about this issue is far from being commonplace, as the opposite scenario has also been reported for nuclear markers [23], [31]–[33]. In particular, Andersen *et al*. [23] suggested the existence of four genetically discrete subpopulations in the Atlantic (*i.e.* West Greenland, NE Atlantic, North Sea and Central North Atlantic) and indicated that this could be the result of the profound ecological differences between feeding areas (environmental conditions, prey availability) posing different selective pressures, coupled with a strong affiliation between mother and calf to the feeding site. Seasonal site fidelity that had been already reported for minke whales [93]–[95] as well as for other species such as humpback whales [96].

We also tested Tiedemann's [97] thesis that states that, for marine large mammals, the F_ST_ obtained for females would reflect the maximum spatial genetic differentiation of the species. Through the population structure observed in the maternally inherited mtDNA, Baker *et al*. [96] demonstrated that humpback whales show strong fidelity to migration destinations such as feeding grounds. Following a similar approach, we performed AMOVA across Management Areas by pooling all females sampled between 2007 and 2011 and, once more, we found no genetic differentiation for microsatellites (F_ST_ = 0.00009, P = 0.326) or mtDNA (F_ST_ = −0.005, P>0.05). This result disagrees, again, with Andersen *et al*. [23] who reported a significant F_ST_ at both markers for females.

In conclusion, our data set of ten microsatellites and 331 bp of mtDNA control region failed to reveal any spatial genetic variation across 2990 individual whales harvested in the five management areas in the NE Atlantic for the years 2004 and 2007–2011.

### Cryptic population genetic structure

The division of North Atlantic minke whale into two mtDNA lineages had already been reported [25], [26], and Palsbøll [26] suggested the presence of two potential breeding populations coexisting at feeding grounds in the North Atlantic. The division of mtDNA showing a lack of concordance between haplotypes and geographic regions was first mentioned by Bakke *et al*. [22] who proposed the existence of two or more differentiated populations sharing the same feeding grounds. However, the pattern observed in mtDNA might also reflect a residual ancestral polymorphism or a “recent” isolation of two populations at breeding sites, which roam through large parts of the North Atlantic Ocean during the feeding migration, as proposed by Palsbøll [26], Bakke *et al*. [22] and Pampoulie *et al*. [25]. Our results also agree with the discordance between haplotypes and geographic areas; however, we support the division of mtDNA into three distinct lineages (Fig. 2), with a central group that evolved through two different expansion episodes. This possible expansion was further corroborated by large negative and significant Fu's Fs, negative Tajima's D (except in 2010), small raggedness values and unimodal mismatch distributions (Table 6). The lack of connections among lineages further suggested genetic differentiation. Importantly, microsatellites did not corroborate this result.

Nuclear markers provided no evidence to reject the hypothesis that North Atlantic minke whales constitute a single panmictic population. This is in spite of certain insights from STRUCTURE analyses conducted both in this study and in Anderwald *et al*. [24] that appeared to spuriously suggest the existence of cryptic subpopulations. First, LnP(D) obtained after the STRUCTURE analyses conducted here revealed that K = 1 was the most likely number of clusters, both for the real data distributed in six year classes and for the 100 simulated panmictic populations. In all cases, a clear decreasing trend of LnP(D) along consecutive values of K was recorded. The *ad hoc* statistic ΔK based on the rate of change in the log probability of data between successive K values obtained through Evanno's test can, unfortunately, never validate K = 1 [58]. Furthermore, this test is not even applicable in situations of decreasing pattern of LnP(D) [58]. However, when ignoring this limitation, Evanno's test showed that the highest ΔK was found at values ranging between K = 2 and K = 4. Thus, in the real data, 5 out of the 6 cases yielded K = 2 at the Evanno criterion, whereas year class 2008 reported K = 3. Likewise, the 100 *in silico* generated panmictic populations revealed K = 2 in a majority of cases (58%) whereas the remaining ones were distributed between K = 3 (33%) and K = 4 (9%). Hence, both LnP(D) pointed at 1, together with the fact that the highest ΔK was found mainly at K = 2 in the real and simulated panmictic populations, supports that North Atlantic minke whale constitutes one single panmictic population.

In addition, when Evanno's test is computed in non-pertinent situations and seems to reveal substructuring in the population (K = 2), there are multiple features that strongly suggest a false result. The first hint to be considered is the low values of ΔK, which is an indication that the strength of the signal detected by STRUCTURE is weak [58]. In our case, both the 100 simulated panmictic populations and the six real ones showed extremely low values of ΔK (ranging mainly from 1 to 10). In contrast, when the differentiation signal between two populations is strong, *i.e.* when the number of clusters is unequivocally two, ΔK exhibits significantly higher values. Thus, for instance, when we conducted STRUCTURE analyses for Atlantic minke whales including Antarctic or Pacific whales as an outgroup (Fig. B in File S1), the value of ΔK at K = 2 was higher by three orders of magnitude than the one found when running STRUCTURE without outgroups. Secondly, when K = 1 but the model is forced for K = 2, most individuals will have a probability around 0.5 and 0.5 of belonging to cluster 1 and cluster 2 respectively. Our results also corroborated this extent as the inferred membership to clusters ranged from 0.51 to 0.64 in the real and the ten *in silico* generated panmictic populations. However, even in this situation, a weak albeit significant F_ST_ between clusters (average value of 0.010 in the real data and of 0.016 in the panmictic populations) can still be found (see Tables A2, A13 in File S1). The fact that values of F_ST_ are of similar magnitude in the real data and in the 100 panmictic populations sheds important doubts about the reliability of such genetic structure.

When running STRUCTURE with outgroups to enhance the genetic differentiation, the resulting barplots for K = 3 (Fig. C in File S1) showed that the subdivision of NE Atlantic minke whales revealed a higher inferred membership to cluster compared to when no outgroups were used. Furthermore, when the outgroup was the Pacific subspecies (*B.a. scammoni*), the averaged inferred membership was 0.89 but when the outgroup was the Antarctic species (*B. bonaerensis*), this value was even higher (average 0.95) and the percentage of non-assigned individuals was slightly lower. This higher inferred membership to cluster could be expected to result in a higher genetic differentiation. However, the resulting F_ST_ values between these clusters were virtually identical in the following cases: real data without outgroups, real data with outgroups, simulated panmictic populations without outgroups, one randomly chosen simulated panmictic population without outgroups (Tables A2, A4, A5, A6, A13 in File S1). Additionally, all of these values overlap with the level of genetic differentiation observed using a similar approach in Anderwald et al. 2011 [24]. The fact that both real and simulated panmictic populations showed the same patterns further increased the doubts upon the reliability of the clustering analyses upon which subdivision of North East Atlantic minke whales into cryptic populations has been suggested [24]. Furthermore, in most cases, the distribution of the individuals belonging to clusters 1 and 2 across Management Areas was surprisingly similar for the six year classes sampled (Table K in File S1), and in the *in silico* generated panmictic populations.

Anecdotally, North Atlantic minke whales have been suggested to follow an annual migration cycle between Arctic feeding grounds and Southern breeding grounds. The information on sightings of minke whales in the Southern North Atlantic is however very scarce [78] and one or more breeding grounds have so far not been demonstrated. Also, foetuses in different stages of development have been found in catches from the northern feeding grounds [78], indicating that mating may take place even there. The hypothesis of panmixia could therefore be well supported by these observations, also implying that separate breeding grounds may not exist.

As a general picture, the data analysed here show that while nuclear markers suggest panmixia, mitochondrial markers reveal the existence of three distinct lineages in North East Atlantic minke whales; which can be a reflection of the different time scales both type of markers represent. Besides, due to maternal inheritance, mitochondrial genes have lower effective migration rates than nuclear genes [98], and random drift is faster for the haploid, maternally inherited mt genome compared to a diploid, biparentally inherited nuclear locus [99]. Furthermore, an accelerated substitution rate of the mitochondrial genome contributes to faster differentiation [100]. Thus, the aforementioned discordance of higher population subdivision in mtDNA than in nuclear DNA is indicative of migration and breeding sex ratios not being biased [101]. Accordingly, and in agreement with Pampoulie *et al*. [25], we did not reach statistical support for the hypothesis of male-biased dispersal in this species. In contrast, male-biased dispersal has been reported for other whale species such as sperm whales [102], [103] or gray whales [86].

## Conclusions

The population structure of North Atlantic minke whale, *B.a. acutorostrata*, has been the subject of a long debate with contrasting results, partially driven by the fact that most previous studies have been limited by low numbers of samples, or genetic markers, or a combination of both. In order to shed further light on this topic, we conducted a spatial, temporal and cryptic population analysis of 2990 whales harvested in the North East Atlantic during the period 2004 and 2007–2011. This large data set, which has been genotyped according to strict protocols upon which the NMDR is based [12], and is thus of very high data quality [36], failed to reveal any indication of geographical or temporal population genetic structure within the NE Atlantic based upon the analysis of ten microsatellites and 331 bp of the mitochondrial D-loop. Furthermore, while three mtDNA lineages were revealed in the data, these did not show any underlying geographic pattern, and possibly represent an ancestral signal. In order to address the possibility of cryptic population structure as suggested by Anderwald *et al*. [24], we run STRUCTURE using a similar approach. However, while Evanno's test might seem to suggest the existence of two genetically differentiated clusters per year class, there were a number of facts strongly suggesting that these results were potentially an artefact. Firstly, as this approach can never validate K = 1, it shows K = 2 instead but with a very low value of ΔK, which is an indication of a very weak genetic signal. Furthermore, there was a lack of corroboration with mtDNA, in each case there was close to a 50/50 division between individuals into groups 1 and 2, and there was an absence of any clear geographic pattern underlying the clusters. The suspicion that these analyses would spuriously reveal population substructure was subsequently confirmed when it was possible to falsely create two cryptic populations in our *in silico* generated panmictic populations. These displayed more or less identical genetic characteristics both as in the real data in this study, and in the study by Anderwald *et al*. [24]. Therefore, we conclude that there is at present no or very little evidence to suggest that the minke whale displays spatial or cryptic population genetic structure throughout the North East Atlantic. However, it is also duly acknowledged that all studies conducted thus far have been limited by low numbers of genetic markers. Therefore, in order to conclusively evaluate the potential for spatial or cryptic population genetic structure within this highly mobile species, significantly larger numbers of markers will be required. Recent publication of the minke whale genome [104] will represent a major resource to identify the numbers of markers needed to address this issue in the future.

## Supporting Information

File S1 **Supporting Information.** File S1 contains detailed information on the following issue: “Testing the hypothesis of cryptic stock clustering in North East Atlantic minke whales”: including Material and Methods, and Results. This appendix also comprises eight figures (Fig. A–G) and thirteen tables (Table A–M). **Figures. Fig. A1.** Bayesian clustering of North East Atlantic minke whales genotyped at 8 microsatellites for the six sampled year classes. Inferred ancestry of individuals was calculated after averaging ten STRUCTURE runs with CLUMPP after Evanno's test. **Fig. A2.** Bayesian clustering of North East Atlantic minke whales genotyped at 10 microsatellites for the six sampled year classes. Inferred ancestry of individuals was calculated after averaging ten STRUCTURE runs with CLUMPP after Evanno's test. **Fig. B.** Bayesian clustering of North East Atlantic minke whale year class 2004 with outgroups: a) 95 individuals of the subspecies Pacific minke whale (*B. a. scammoni*); b) 93 individuals of the Antarctic minke whale (*B. bonaerensis*), and c) both former outgroups together. The number of clusters that best fitted the data was K = 2 after Evanno's [58] test in each case. This scenario was consistent across year classes. **Fig. C.** Bayesian clustering of North East Atlantic minke whale with outgroups in each year class. In the column to the left, the outgroup are 95 individuals of the subspecies Pacific minke whale (*B. a. scammoni*) whereas in the column to the right, the outgroup are 93 individuals of the Antarctic minke whale (*B. bonaerensis*). The number of clusters that best fitted the data was distinctively K = 2 after Evanno's [58] test in each case. **Fig. D.** Geographic distribution of individuals after different clustering methods: BAPS and STRUCTURE for microsatellites. Pie charts represent the percentage of individuals belonging to clusters 1 (dark grey) and 2 (light grey) per Management Area taking year class 2008 as an example (the full data for all the year classes is available in Table K in File S1). **Fig. E.** Bayesian clustering of individuals of ten of the simulated panmictic populations that showed K = 2 after Evanno's test. Inferred ancestry of individuals was calculated after averaging ten STRUCTURE runs with CLUMPP. **Fig. F.** Distribution of pairwise F_ST_ after 10000 random clustering of North Atlantic minke whale individuals per year class into two groups. **Fig. G.** Frequency distributions of the corrected assignment index (AIc) for 2156 females (light grey bars above axis) and 834 males (dark grey bars below axis). AIc values differed among sexes, males having on average negative values (−0.051) and higher variance (2.90) and females positive values (0.020) with lower variance (2.35). However, Mann–Whitney U-test proved sex-biased dispersal to be non-significant (*P*>0.5). **Tables. Table A.** Summary result of STRUCTURE without outgroups: a) Data set of 8 microsatellites. b) Data set of 10 microsatellites. **Table B.** STRUCTURE without outgroups: Clustering of individuals per year class after Evanno's test (the two cases that showed the highest Evanno's ΔK at K = 3 are depicted in italics and analysed for K = 2 for comparison): Number of individuals per cluster and range of inferred membership to each of them (in brackets). Summary of the results of the AMOVA (F_ST_ and *P*-value) conducted with Arlequin with 10000 permutations. Analyses were performed for the same sets of individuals genotyped at mtDNA. Statistically significant values were highlighted in boldface type. Negative F_ST_ values found at mtDNA were transformed into 0. a) Data set of 8 microsatellites. b) Data set of 10 microsatellites. **Table C.** Summary statistics after STRUCTURE clustering showing total number of alleles, number of private alleles, observed heterozygosity (average ± SE), unbiased expected heterozygosity (average ± SE), and inbreeding coefficient (F_IS_) (average ± SD). We show in italics the distribution of the individuals for K = 2 for the two year classes that showed the highest Evanno's ΔK at K = 3. a) Data set of 8 microsatellites. b) Data set of 10 microsatellites. **Table D.** STRUCTURE with the Pacific minke whale subspecies (*B. a. scammoni*) as an outgroup. Clustering of individuals per year class and one randomly chosen simulated panmictic population after Evanno's test and CLUMPP averaging: Number of individuals per cluster and range of inferred membership to each of them (in brackets). Summary of the results of the AMOVA (F_ST_ and *P*-value) conducted with Arlequin with 10000 permutations. Analyses were performed for the same sets of individuals genotyped at mtDNA. Statistically significant values were highlighted in boldface type. Negative F_ST_ values found at mtDNA were transformed into 0. **Table E.** STRUCTURE with Antarctic minke whale species (*B. bonaerensis*) as an outgroup. Clustering of individuals per year class and one randomly chosen simulated panmictic population after Evanno's test and CLUMPP averaging: Number of individuals per cluster and range of inferred membership to each of them (in brackets). Summary of the results of the AMOVA (F_ST_ and *P*-value) conducted with Arlequin with 10000 permutations. Analyses were performed for the same sets of individuals genotyped at mtDNA. Statistically significant values were highlighted in boldface type. Negative F_ST_ values found at mtDNA were transformed into 0. **Table F.** STRUCTURE consensus clustering of individuals (*i.e.* agreement between Antarctic and Pacific outgroup clustering) into two groups per year class. Summary of the results of the AMOVA (F_ST_ and *P*-value) conducted with Arlequin with 10000 permutations. Analyses were performed for the same sets of individuals at mtDNA. Statistically significant values are highlighted in boldface type. **Table G.** Summary statistics after STRUCTURE consensus clustering (*i.e.* consensus between Antarctic and Pacific outgroup clustering) showing total number of alleles, allelic richness (minimum sample size), number of private alleles, observed heterozygosity (average ± SE), unbiased expected heterozygosity (average ± SE), and inbreeding coefficient (F_IS_) (average ± SD). **Table H.** BAPS clustering of individuals genotyped with microsatellites into two groups per year class. Summary of the results of the AMOVA (F_ST_ and *P*-value) conducted with ARLEQUIN with 10000 permutations. Analyses were performed for the same sets of individuals at mtDNA. Statistically significant values were highlighted in boldface type. **Table I.** Summary statistics after BAPS clustering showing total number of alleles, allelic richness (minimium sample size), number of private alleles, observed heterozygosity (average ± SE), unbiased expected heterozygosity (average ± SE), and inbreeding coefficient (F_IS_) (average ± SD). **Table J.** GeneClass self-assignment: Percentage of individuals genotyped at microsatellites that were correctly assignment after clustering procedures. **Table K.** Number of individuals genotyped at microsatellites per Management Areas after clustering with BAPS and STRUCTURE (with and without outgroup). ND = No data. **Table L.** Matrix of numbers and percentage of coincident individuals when comparing the three clustering methods: BAPS, STRUCTURE without outgroup (STR), and STRUCTURE with outgroup (STR consensus). The percentage of coincident individuals was calculated by dividing the number of by the lowest number of individuals in the corresponding cluster. STRUCTURE analyses were performed with 8 microsatellites. **Table M**. STRUCTURE clustering of individuals in the 10 randomly selected simulated panmictic populations showing K = 2 after Evanno's test. Number of individuals per cluster and range of inferred membership to each of them (in brackets); number of non-assigned individuals (and % of the total). Summary of the results ofthe AMOVA (F_ST_ and *P*-value) conducted with Arlequin with 10000 permutations. Statistically significant values were highlighted in boldface type.
(DOCX)
