# Supplementary material for: Investigating Population Genetic Structure in a Highly Mobile Marine Organism: The Minke Whale Balaenoptera acutorostrata acutorostrata in the North East Atlantic
Source: PLoS One. 2014 Sep 30;9(9):e108640. doi: 10.1371/journal.pone.0108640 (PMC4182549; doi:10.1371/journal.pone.0108640)
Supplement: File S1 — Supporting Information. File S1 contains detailed information on the following issue: “Testing the hypothesis of cryptic stock clustering in North East Atlantic minke whales”: including Material and Methods, and Results. This appendix also comprises eight figures (Fig. A–G) and thirteen tables (Table A–M). Figures. Fig. A1. Bayesian clustering of North East Atlantic minke whales genotyped at 8 microsatellites for the six sampled year classes. Inferred ancestry of individuals was calculated after averaging ten STRUCTURE runs with CLUMPP after Evanno's test. Fig. A2. Bayesian clustering of North East Atlantic minke whales genotyped at 10 microsatellites for the six sampled year classes. Inferred ancestry of individuals was calculated after averaging ten STRUCTURE runs with CLUMPP after Evanno's test. Fig. B. Bayesian clustering of North East Atlantic minke whale year class 2004 with outgroups: a) 95 individuals of the subspecies Pacific minke whale (B. a. scammoni); b) 93 individuals of the Antarctic minke whale (B. bonaerensis), and c) both former outgroups together. The number of clusters that best fitted the data was K = 2 after Evanno's [58] test in each case. This scenario was consistent across year classes. Fig. C. Bayesian clustering of North East Atlantic minke whale with outgroups in each year class. In the column to the left, the outgroup are 95 individuals of the subspecies Pacific minke whale (B. a. scammoni) whereas in the column to the right, the outgroup are 93 individuals of the Antarctic minke whale (B. bonaerensis). The number of clusters that best fitted the data was distinctively K = 2 after Evanno's [58] test in each case. Fig. D. Geographic distribution of individuals after different clustering methods: BAPS and STRUCTURE for microsatellites. Pie charts represent the percentage of individuals belonging to clusters 1 (dark grey) and 2 (light grey) per Management Area taking year class 2008 as an example (the full data for all the year classes is a [file pone.0108640.s001.docx]

**File S1. Supporting Information**

**Testing the hypothesis of cryptic stock clustering in North East Atlantic minke whales**

María Quintela^1,2^, Hans J. Skaug^1,3^, Nils Øien^1^, Tore Haug^4^, Bjørghild B. Seliussen^1^, Hiroko K. Solvang^1^, Christophe Pampoulie^6^, Naohisa Kanda^7^, Luis A. Pastene^7^, Kevin A. Glover^1,8^

**MATERIAL AND METHODS**

**Testing the hypothesis of cryptic stock clustering in North East Atlantic minke whale**

Genetic clusters were identified using STRUCTURE v. 2.3.4 [[1-3](#_ENREF_1)] under a model assuming admixture and correlated allele frequencies without using population information. Ten runs with a burn-in period consisting of 100000 replications and a run length of 1000000 MCMC iterations were performed for K1 to K6. If applicable, we used STRUCTURE Harvester [[4](#_ENREF_4)] to calculate the Evanno *et al*. [[5](#_ENREF_5)] *ad hoc* summary statistic ΔK. Runs were automatized with ParallelStructure [[6](#_ENREF_6)], averaged with CLUMPP [[7](#_ENREF_7)] and graphically displayed using barplots. This approach was followed both using outgroups (*B.a. scammoni* and *B. bonaerensis*) and without them. It should be noted that the data set for the outgroups was only genotyped at eight of the ten microsatellite loci; therefore, results for STRUCTURE without outgroups are also provided for eight markers to ease the comparisons.

We also tested BAPS 6.0 [[8](#_ENREF_8)] for two subpopulations and thus we run the program 100 times for K=2 and performed the most likely admixture of genotypes [[9](#_ENREF_9)] on a per year class basis.

Genetic differentiation between individuals distributed by the resulting BAPS and STRUCTURE clustering approaches respectively was further tested using both AMOVA with 10000 permutations implemented in ARLEQUIN v.3.5.1.2 [[10](#_ENREF_10)], and Fisher’s exact tests [[11](#_ENREF_11)] with Markov chain settings of 10000 steps of dememorisation, 1000 batches and 10000 iterations per batch implemented in GENEPOP on the web [[12](#_ENREF_12)]. Finally, a Factorial Correspondence Analysis (FCA) was applied using the software GENETIX [[13](#_ENREF_13)] to further assess the distribution of samples by putative population based on individual genotypes.

To gain some insight into the strength of the genetic differentiation between the groups generated with the abovementioned clustering methods, we repeatedly split the data in two random groups of even size for each year class. Pairwise F_ST_ [[14](#_ENREF_14)] was computed between groups 10000 times. The obtained distribution of F_ST_ was plotted, which represents the null distribution under the hypothesis that group assignment is a random process. Finally, the higher 95^th^ higher percentile was compared with the F_ST_ obtained after BAPS and STRUCTURE clustering.

An individual-based assignment test, implemented in the program GeneClass 2.0 [[15](#_ENREF_15),[16](#_ENREF_16)] was performed to validate STRUCTURE and BAPS clustering using a Bayesian assignment approach [[17](#_ENREF_17)].

The groups resulting after clustering methods based on microsatellites were replicated with the same individuals genotyped at mtDNA and subjected to AMOVA to calculate the pairwise F_ST_ both by taking into account haplotype frequencies and also the genetic distance between haplotypes using the Tamura and Nei’s [[18](#_ENREF_18)] genetic distance model. Significance was assessed after 10000 permutations.

Furthermore, to formally test the hypothesis of minke whale constituting a panmictic population, we used the data from year 2007 to create a set of 100 simulated panmictic populations. At each of the ten loci, the allelic values (two per individual) were put in a pool, and then randomly re-assigned to individuals, thereby preserving the original allele frequencies. The 100 simulated populations were analysed automatizing STRUCTURE without outgroups under the same conditions of the real data. Even if not applicable in this situation, Evanno’s test was conducted for the 100 simulated populations. Ten of them yielding K=2 after Evanno test, were averaged with CLUMPP and pairwise F_ST_ between resulting clusters was performed as above. In addition, BAPS analyses were also conducted for a range of K between 1 and 5.

This simulation (permutation) procedure allowed a *p*-value for the Evanno-K test statistic to be evaluated under the null hypothesis of the population being panmictic. The *p*-value is simply the proportion of 100 simulated K’s exceeding (or equal to) the Evanno-K found in the real 2007 data set.

**RESULTS**

**Testing the hypothesis of cryptic stock clustering of North East Atlantic minke whale**

The results of STRUCTURE clustering both using outgroups and without them are as follows:

1. STRUCTURE without outgroup (Fig. A1, A2).- The highest average likelihood was distinctively found at K=1 in all year classes, together with a decreasing trend of LnP(D) across consecutive values of K (Table Aa,b). In these situations, although Evanno’s test is not applicable, we conducted it anyhow and found that ΔK took its highest value for K=2 in all the year classes but 2008 (K=3). The common feature to all the sampling years were the low values reported for ΔK, which reached its maximum in 2011 (ΔK=16.7). At K=2, the number of individuals per cluster was even per year class with a ratio ranging between of 1.0-1.3 (Table Ba,b), very narrow ranges of membership (0.51-0.63, average 0.59) and a percentage of unassigned individuals ranging between 8.7 and 18.7%. Fisher’s exact test showed a clear separation between groups ($\chi$^2^=infinity, df=20, *P*<0.0001) per year class and the Factorial Correspondence Analyses further confirmed it (despite a low percentage of the total variation explained by the two first axes, which ranged between 3.97 and 4.22%). Interestingly, the same individuals genotyped at mtDNA did not produce any significant F_ST_ in any sampling year (Table B a,b). F_ST_ after STRUCTURE clustering was significantly higher than the ones obtained after random clustering of individuals (Table B a,b; Fig. F). STRUCTURE-cluster 2, the one holding lower number of individuals per year, also consistently showed higher number of private alleles, and also higher number of alleles (in five and six out of six cases) whereas Ho, uHe and F_IS_ took similar values in both clusters (Table A3a,b). The assignment of individual microsatellite genotypes to their most likely source population using GeneClass corroborated STRUCTURE clustering with an average percentage of correct assignment of 86% (ranging from 83.5 to 90.2%, Table J).
2. STRUCTURE with outgroups (Fig. C).- In these cases, Evanno´s test was indeed pertinent and showed K=2 in all the combinations of analyses with outgroups (*i.e.* either using one outgroup or the two of them simultaneously). Hence, with only one outgroup (either *B.a. scammoni* or *B. bonaerensis*), both NE Atlantic minke whales (*B.a. acutorostrata*) and the outgroup constituted two very distinct and compact clusters. However, when the two outgroups were included at the same time, NE Atlantic minke whale individuals (*B.a. acutorostrata*) clustered in a compact group separated from all the rest (*B.a. scammoni* and *B. bonaerensis*) that gathered in another very compact one (barplots for year class 2004 are shown as an example in Fig. 2). This scenario was consistent over the six year classes and the randomly chosen simulated population. When using outgroups, we needed to explore K=3 to have *B.a. acutorostrata* minke whales divided into two groups (*e.g.* Fig. 2 c-f) and the partition of individuals into clusters was conditioned to overcoming a threshold of membership of 0.50. When the Antarctic minke whale (*B. bonaerensis*) was the outgroup, the percentage of North Atlantic individuals (*B.a. acutorostrata*) not assigned to any cluster (*i.e.* not overcoming the 0.50 threshold) ranged between 0.9 and 3%; however, when using the Pacific subspecies (*B.a. scammoni*), the percentage of un-assignment slightly raised (1.5-5.2%) (Table D, A5).
   1. Outgroup Pacific minke whale (*B.a. scammoni*) (Fig. C left column).- The average membership to cluster was 0.89 and the average F_ST_ found between clusters was 0.0137. The same individuals genotyped at mtDNA did not show any kind of genetic differentiation between clusters (Table D).
   2. Outgroup: Antarctic minke whale (*B. bonaerensis*) (Fig. C right column).-The average membership to cluster was higher, 0.95, whereas the average F_ST_ between clusters was slightly slower, 0.0122. Again, the individuals from both clusters genotyped at mtDNA did not reveal any genetic differentiation (Table E)
   3. A more conservative alternative could be chosen, *i.e.* dividing the NE Atlantic minke whales into two groups after consensus the results of the former analyses. This consensus meant that individuals were assigned to cluster 1 or 2 after comparing the assignment obtained after Antarctic and Pacific analyses. Likewise, a number of individuals was left unassigned and this comprised those that did not reach the inferred ancestry 0.5 threshold plus the mismatches between both procedures (*e.g.* individuals that belonged to cluster 1 with Antarctic outgroup and to cluster 2 in the Pacific clustering). The percentage of coincidence of individuals per cluster and year class ranged between 84 and 95% (Table F) and left some 3-9% of non-assigned individuals. The conjunct use of both outgroups did not improve the clustering whatsoever and therefore it was not further considered.

The consensus clustering of NE Atlantic minke whale yielded two groups of even size on a year class basis that showed significant F_ST_ and R_ST_ per year, but R_ST_ in 2007 (Table F) and also a clear separation between groups according to Fisher’s exact test ($\chi$^2^=infinity, df=20, *P*<0.0001). Likewise, the Factorial Correspondence Analyses further confirmed the separation between clusters despite the low percentage of the total variation explained by the two first axes that ranged between 3.9 and 4.2%. Once more, the same individuals genotyped at mtDNA did not show any evidence of genetic differentiation (Table F). F_ST_ after STRUCTURE consensus clustering was again significantly higher than the ones obtained after random clustering of individuals (Table F, Fig. F). The total number of alleles per cluster was quite even but yet slightly higher for the smallest cluster in four out of six of the comparisons (Table G); and likewise, the number of private alleles. Allelic richness, Ho, uHe and F_IS_ took similar values in both clusters. The assignment of individual microsatellite genotypes to their most likely source population using GeneClass corroborated STRUCTURE consensus clustering with a high percentage of correct assignment (97-98.6%) across all year classes but 2008 (85.6%), Table J.

BAPS divided individuals of each sampling year class into two groups of even size for year classes 2008, 2009 and 2011 whereas for the remaining ones, the size ratio was around 1.4-1.5 (Table H). No admixed individuals were detected in any of the sampling years. This approach of clustering revealed weak, albeit significant, F_ST_ in each case that ranged from 0.0059 to 0.0151 (*P*<0.0001); and likewise, significant R_ST_ (ranging from 0.0033 to 0.0223). These F_ST_ values were again significantly higher than the ones obtained after random clustering of individuals (Table H, Fig. F). Fisher’s exact test also showed a clear separation between clusters ($\chi$^2^=infinity, df=20, *P*<0.0001) per year class; which was further confirmed by the Factorial Correspondence Analyses despite the low percentage of the total variation explained by the two first axes (between 3.73 and 4.10%). The same individuals genotyped at mtDNA did not produce significant F_ST_ with the exception of year classes 2007 and 2008 based on Tamura-Nei distance and haplotype frequencies, respectively (Table H). BAPS-cluster 1, the one holding the highest number of individuals per year, also consistently showed higher number of total and private alleles whereas allelic richness, Ho, uHe and F_IS_ took similar values in both clusters (Table I).The assignment of individual microsatellite genotypes to their most likely source population using GeneClass corroborated BAPS clustering with a percentage of correct assignment of 100% in all the cases (Table J).

In general, any type of STRUCTURE clustering revealed slightly stronger F_ST_ per year class than BAPS (except in 2011) (Tables A2, A4, A5, A6). The F_ST_ obtained after random clustering was always lower than the F_ST_ obtained either after BAPS or STRUCTURE clustering for the same year (Fig. F)

The geographic distribution of individuals after both procedures of clustering was slightly different: STRUCTURE-clustered individuals were evenly distributed among Management Areas in each year class whereas for the BAPS-clustered ones, this distribution was less homogeneous in some of the cases (Fig. D and Table K).

The matrix in Table L reports the accuracy of the clustering procedures for individuals genotyped at microsatellites and summarizes the number and percentage of coincidences of the pairwise comparisons after the three clustering methods. The comparison BAPS *vs*. STRUCTURE consensus clustering showed the highest coincidence: average of 74% (ranging from 54 to 90). On the other hand, the comparison between STRUCTURE methods (i.e. with *vs*. without outgroups) showed the lowest degree of accuracy: an average of 55% of coincident individuals (ranging between 46 and 67%).

The analyses of the simulated panmictic populations in STRUCTURE (*i.e.* without outgroups) showed that the highest average likelihood was found for K=1 in all 100 cases, together with a decreasing trend of LnP(D) across consecutive K; as it had been formerly found for the real data. Again, although this is a typical situation for Evanno’s test not being applicable, we conducted it anyhow to assess the proportions of the different solutions it provided. Hence, in 58% of the cases, the most likely number of clusters was K=2, followed by K=3 in 33% and K=4 in the remaining 9%. As observed in the real data, low values of ΔK were reported, ranging from 1 till again a maximum of 15. Because all simulated data sets had K≥2, *i.e.* larger or equal to the K=2 which was obtained for the real data, we obtained a *P*-value of 1.00 and hence we cannot reject the null hypothesis of a panmictic population. CLUMPP was performed on ten randomly chosen populations that showed K=2 after Evanno’s test and individuals were distributed into clusters after overcoming a threshold of 0.50. In all cases, both clusters showed similar size (ratio 1-1.3) and 7-19% of individuals were left unassigned (Table M). Pairwise F_ST_ computed between the groups in which these panmictic populations were divided revealed highly significant genetic structure that ranged between 0.012 and 0.020 (*P*<0.0001). BAPS revealed that, in spite of dealing with panmictic populations, in no case the most likely K was 1. Instead, the number of putative populations took the following values: 3 (4% of the cases), 4 (38%) and 5 (58%).

**REFERENCES**

1. Falush D, Stephens M, Pritchard JK (2003) Inference of population structure using multilocus genotype data: Linked loci and correlated allele frequencies. Genetics 164: 1567-1587.

2. Pritchard JK, Stephens M, Donnelly P (2000) Inference of population structure using multilocus genotype data. Genetics 155: 945-959.

3. Hubisz M, Falush D, Stephens M, Pritchard J (2009) Inferring weak population structure with the assistance of sample group information. Molecular Ecology Resources 9: 1322 - 1332.

4. Earl DA, von Holdt BM (2012) STRUCTURE HARVESTER: a website and program for visualizing STRUCTURE output and implementing the Evanno method. Conservation Genetics Resources 4: 359-361.

5. Evanno G, Regnaut S, Goudet J (2005) Detecting the number of clusters of individuals using the software STRUCTURE: a simulation study. Molecular Ecology 14: 2611-2620.

6. Besnier F, Glover KA (2013) ParallelStructure: a R package to distribute parallel runs of the population genetics program STRUCTURE on multi-core computers. PLoS ONE 8: e70651.

7. Jakobsson M, Rosenberg NA (2007) CLUMPP: a cluster matching and permutation program for dealing with label switching and multimodality in analysis of population structure. Bioinformatics 23: 1801-1806.

8. Corander J, Waldmann P, Marttinen P, Sillanpaa MJ (2004) BAPS 2: enhanced possibilities for the analysis of genetic population structure. Bioinformatics 20: 2363-2369.

9. Corander J, Waldmann P, Sillanpaa MJ (2003) Bayesian analysis of genetic differentiation between populations. Genetics 163: 367-374.

10. Excoffier L, Laval G, Schneider S (2005) Arlequin ver. 3.0: An integrated software package for population genetics data analysis. Evolutionary Bioinformatics Online 1: 47-50.

11. Rousset F, Raymond M (1995) Testing heterozygote excess and deficiency. Genetics 140: 1413-1419.

12. Rousset F (2008) GENEPOP'007: a complete re-implementation of the genepop software for Windows and Linux. Molecular Ecology Resources 8: 103-106.

13. Belkhir K, Borsa P, Chikhi L, Raufaste N, Bonhomme F (2004) GENETIX 4.05, logiciel sous Windows TM pour la génétique des populations. Montpellier (France): Laboratoire Génome, Populations, Interactions, CNRS UMR. Université de Montpellier II.

14. Weir BS, Cockerham CC (1984) Estimating F-statistics for the analysis of population structure. Evolution 38: 1358-1370.

15. Cornuet J-M, Piry S, Luikart G, Estoup A, Solignac M (1999) New methods employing multilocus genotypes to select or exclude populations as origins of individuals. Genetics 153: 1989-2000.

16. Piry S, Alapetite A, Cornuet J-M, Paetkau D, Baudouin L, et al. (2004) GENECLASS2: A software for genetic assignment and first-generation migrant detection. Journal of Heredity 95: 536-539.

17. Rannala B, Mountain JL (1997) Detecting immigration by using multilocus genotypes. Proceedings of the National Academy of Sciences 94: 9197-9201.

18. Tamura K, Nei M (1993) Estimation of the number of nucleotide substitutions in the control region of mitochondrial DNA in humans and chimpanzees. Molecular Biology and Evolution 10: 512-526.

**FIGURES**

| **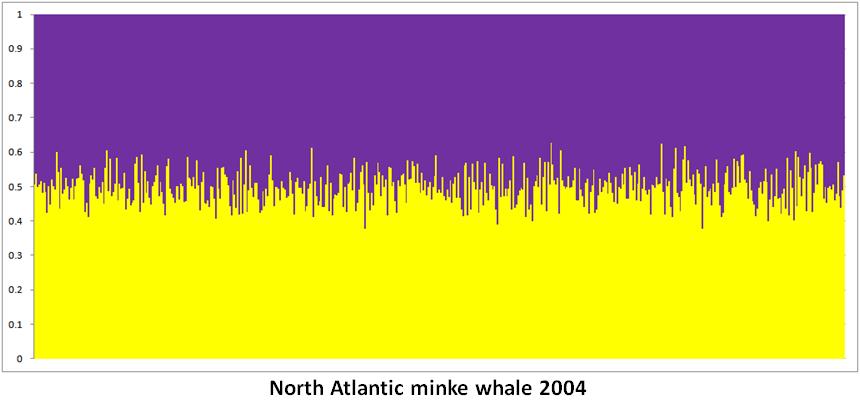** | **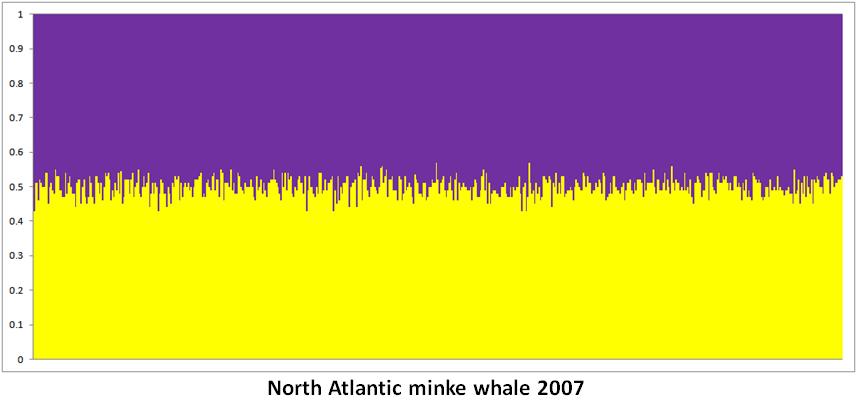** |
| --- | --- |
| **** | **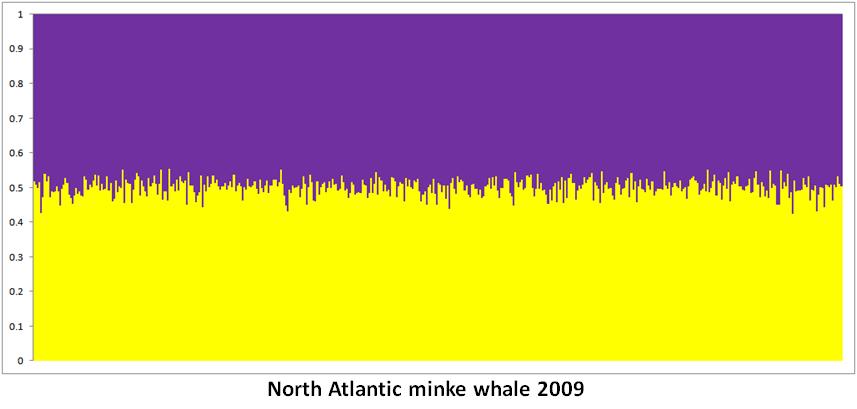** |
| **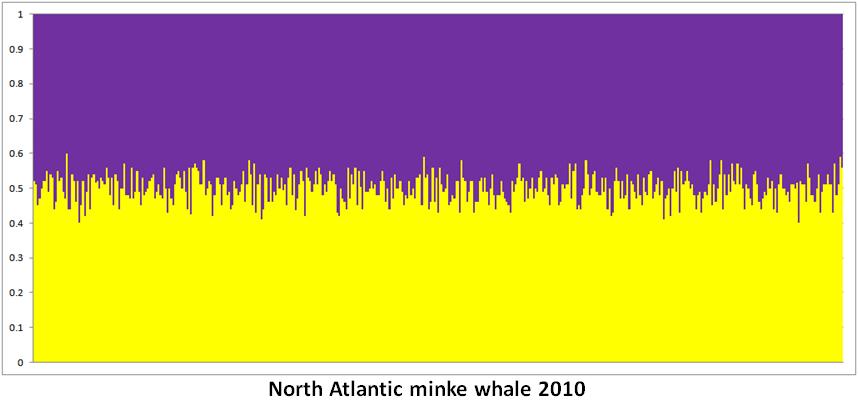** | **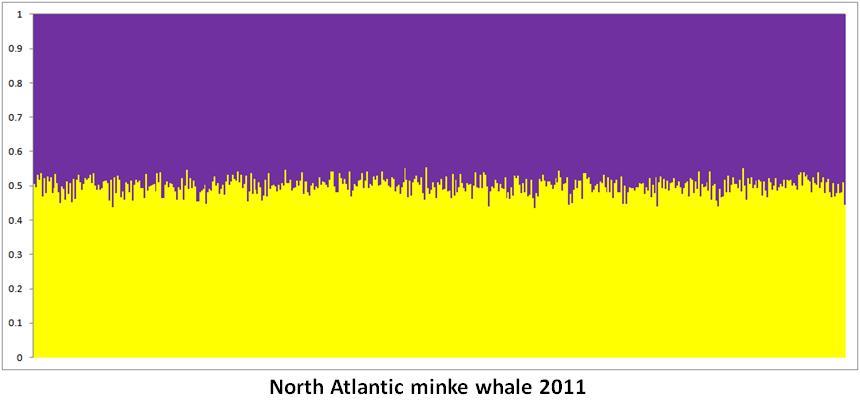** |

**Fig. A1.** Bayesian clustering of North East Atlantic minke whales genotyped at 8 microsatellites for the six sampled year classes. Inferred ancestry of individuals was calculated after averaging ten STRUCTURE runs with CLUMPP after Evanno’s test.

| **** | **** |
| --- | --- |
| **** | **** |
| **** | **** |

**Fig. A2.** Bayesian clustering of North East Atlantic minke whales genotyped at 10 microsatellites for the six sampled year classes. Inferred ancestry of individuals was calculated after averaging ten STRUCTURE runs with CLUMPP after Evanno’s test.

| a) 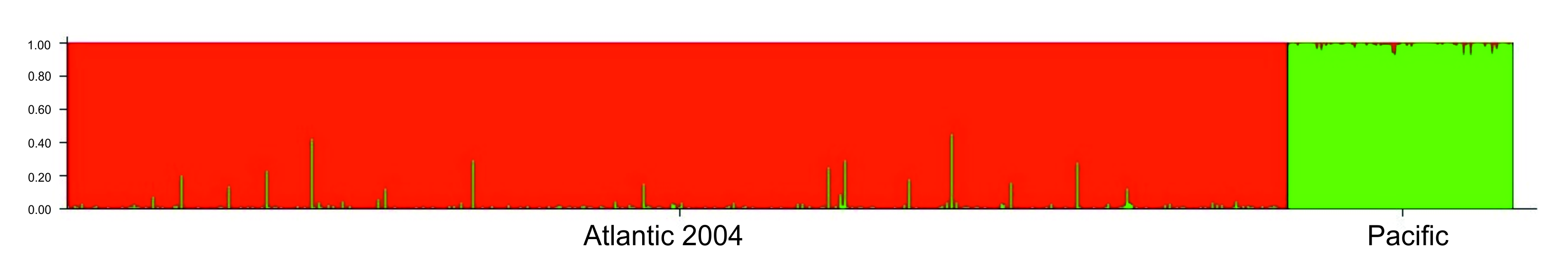 |
| --- |
| b) 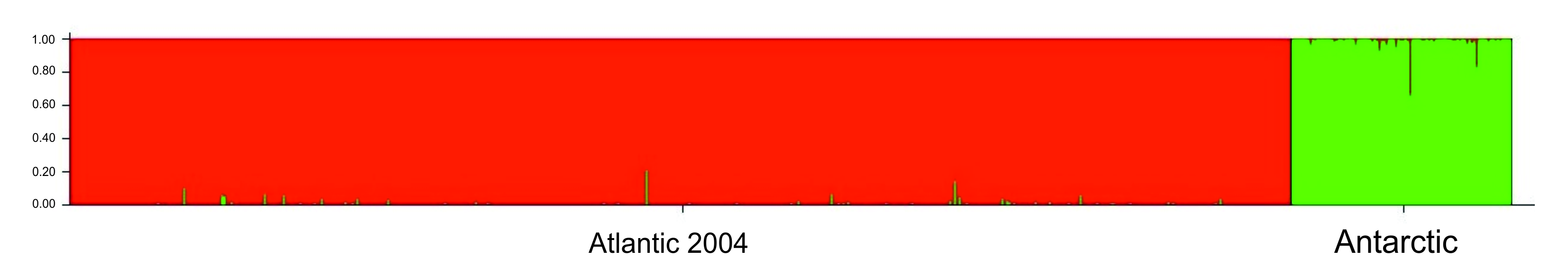 |
| c) 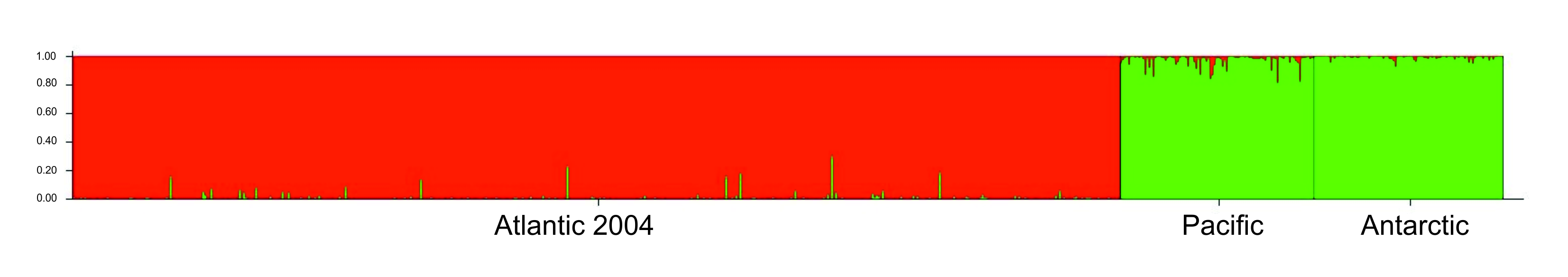 |

Fig. B. Bayesian clustering of North East Atlantic minke whale year class 2004 with outgroups: a) 95 individuals of the subspecies Pacific minke whale (*B. a. scammoni*); b) 93 individuals of the Antarctic minke whale (*B. bonaerensis*), and c) both former outgroups together. The number of clusters that best fitted the data was K=2 after Evanno’s [[5](#_ENREF_5)] test in each case. This scenario was consistent across year classes.

| 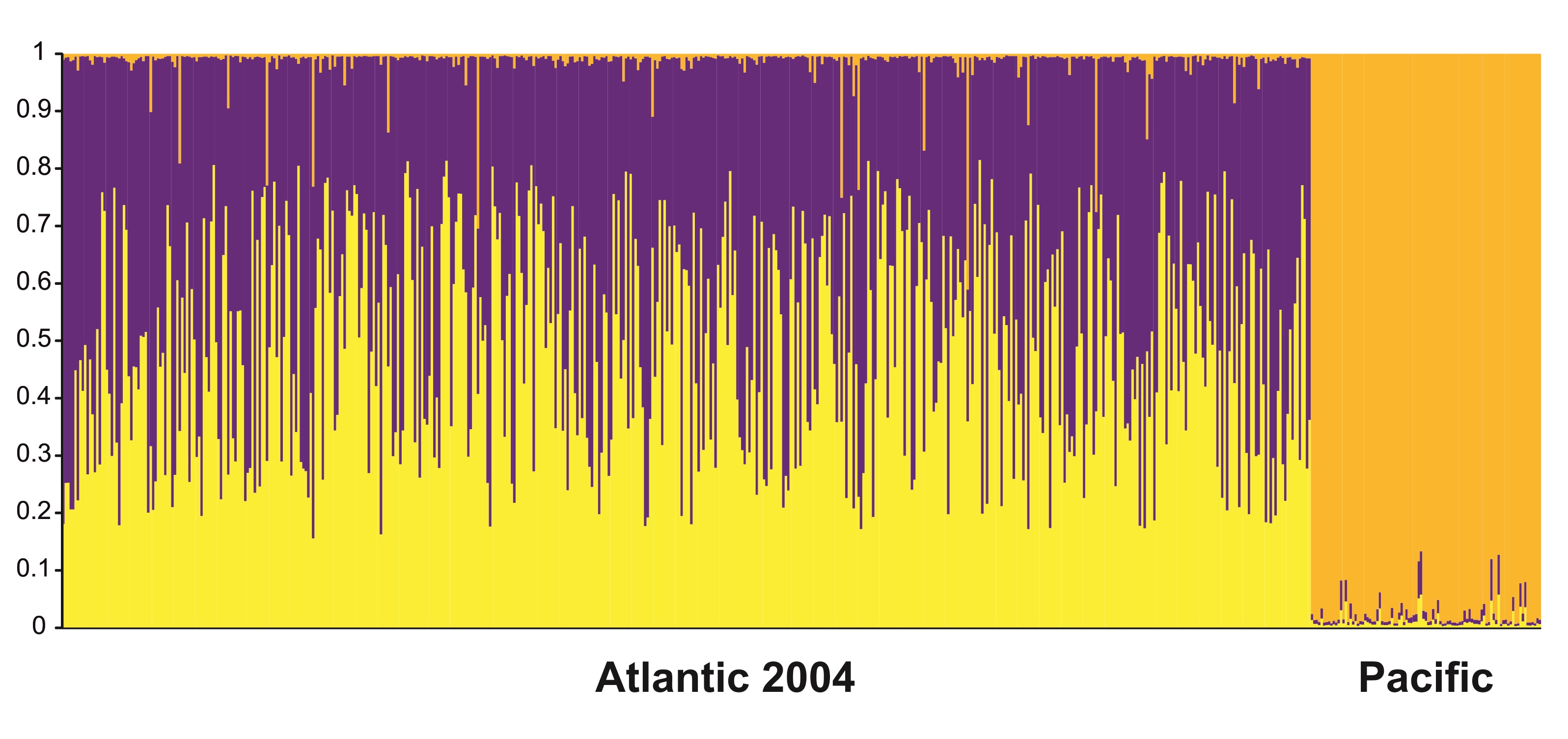 | 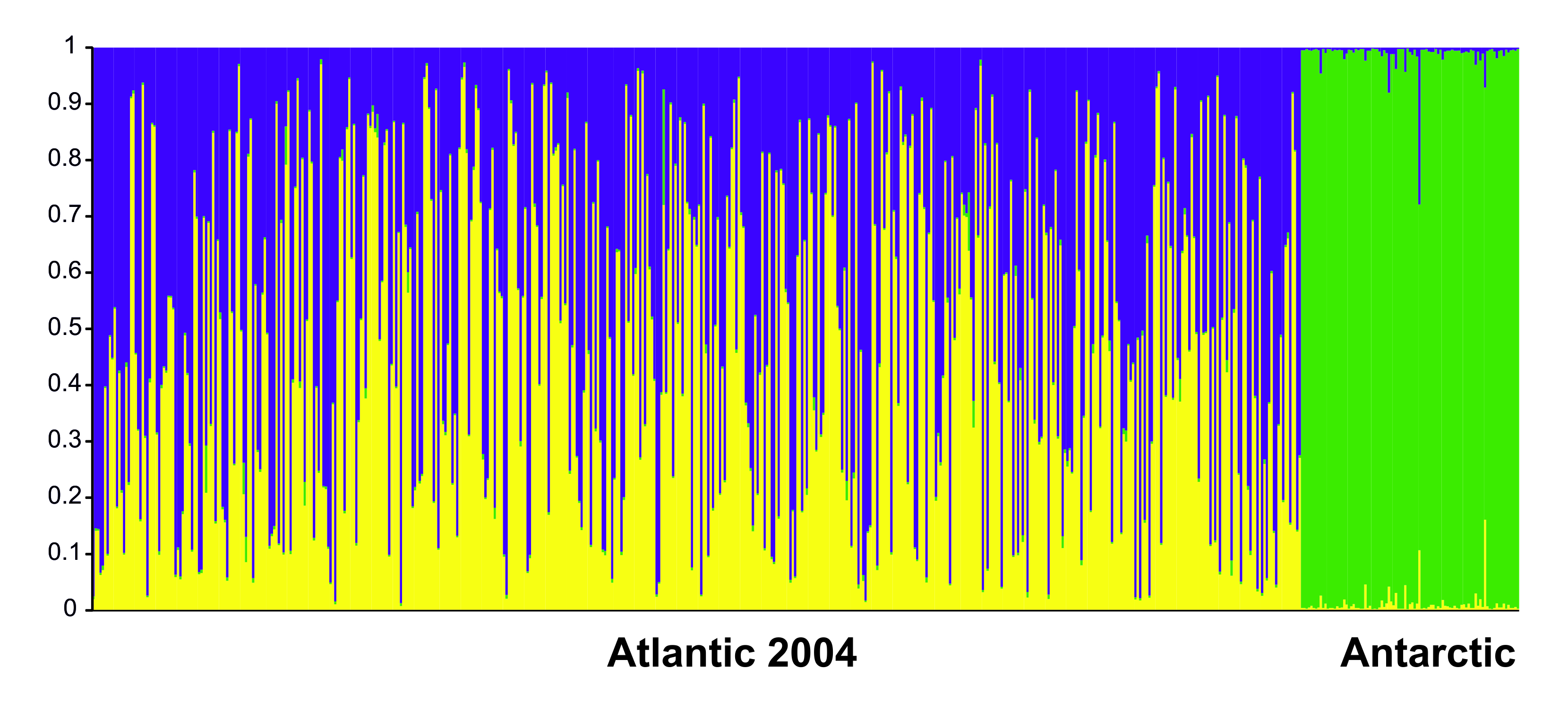 |
| --- | --- |
| 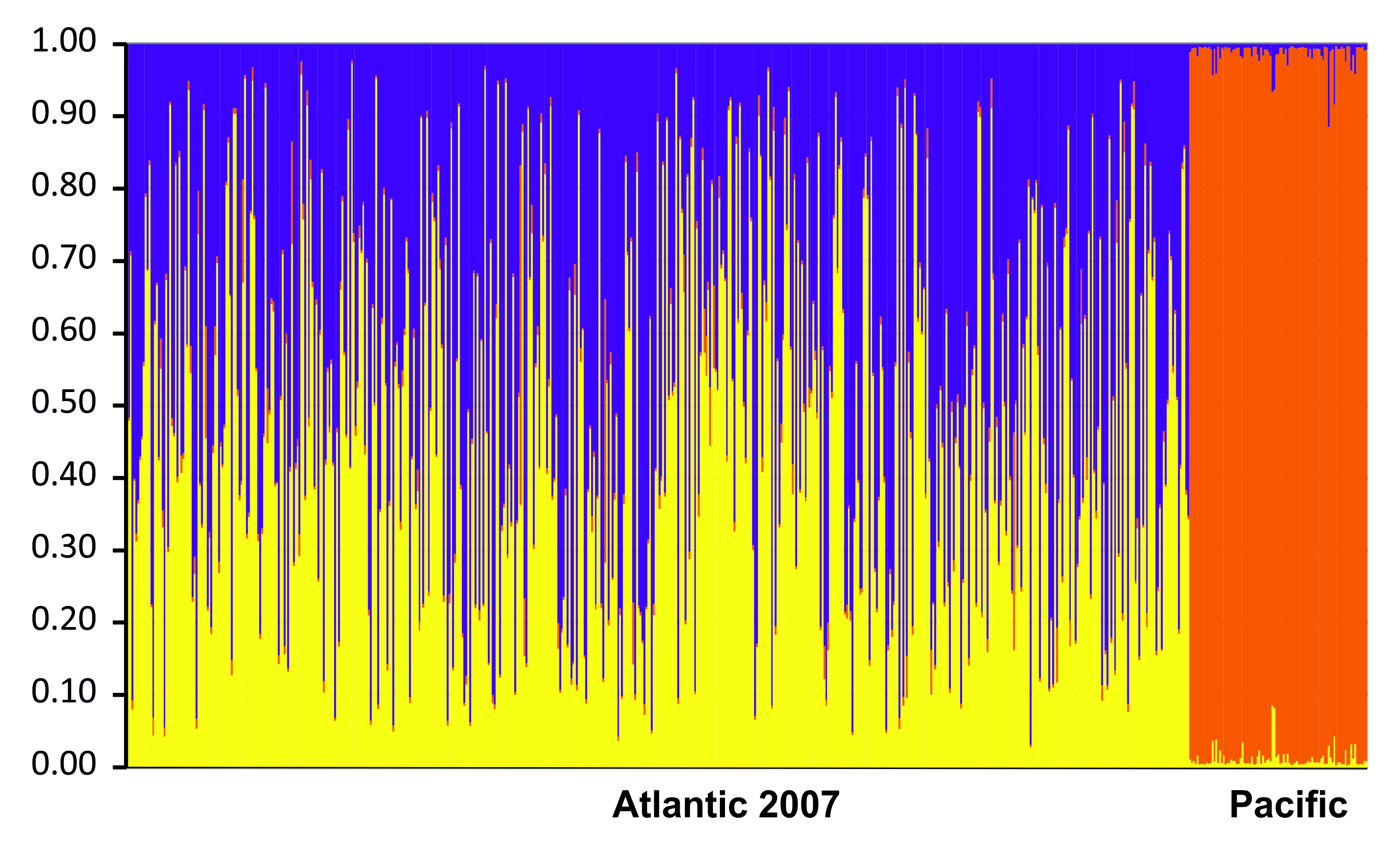 | 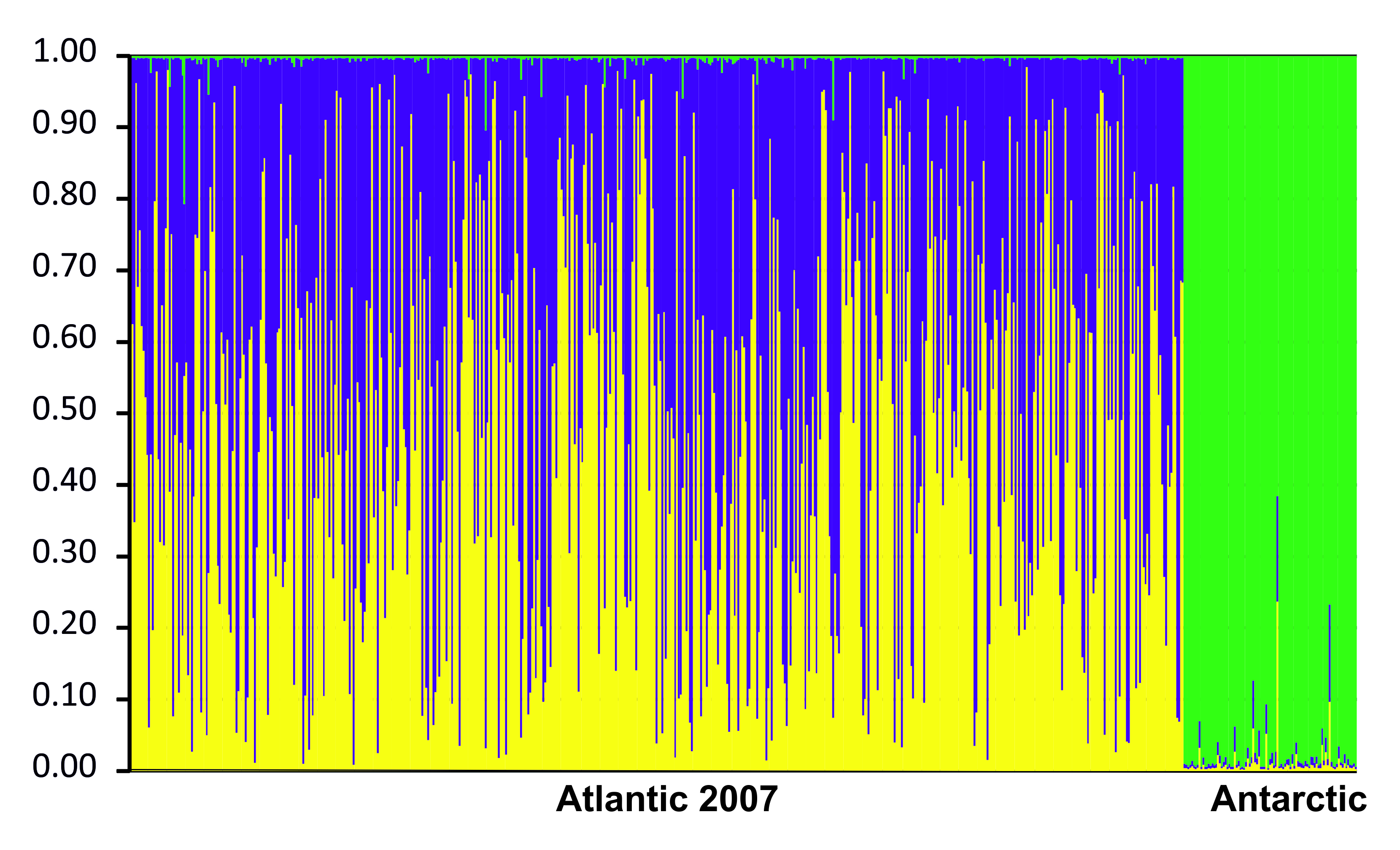 |
| 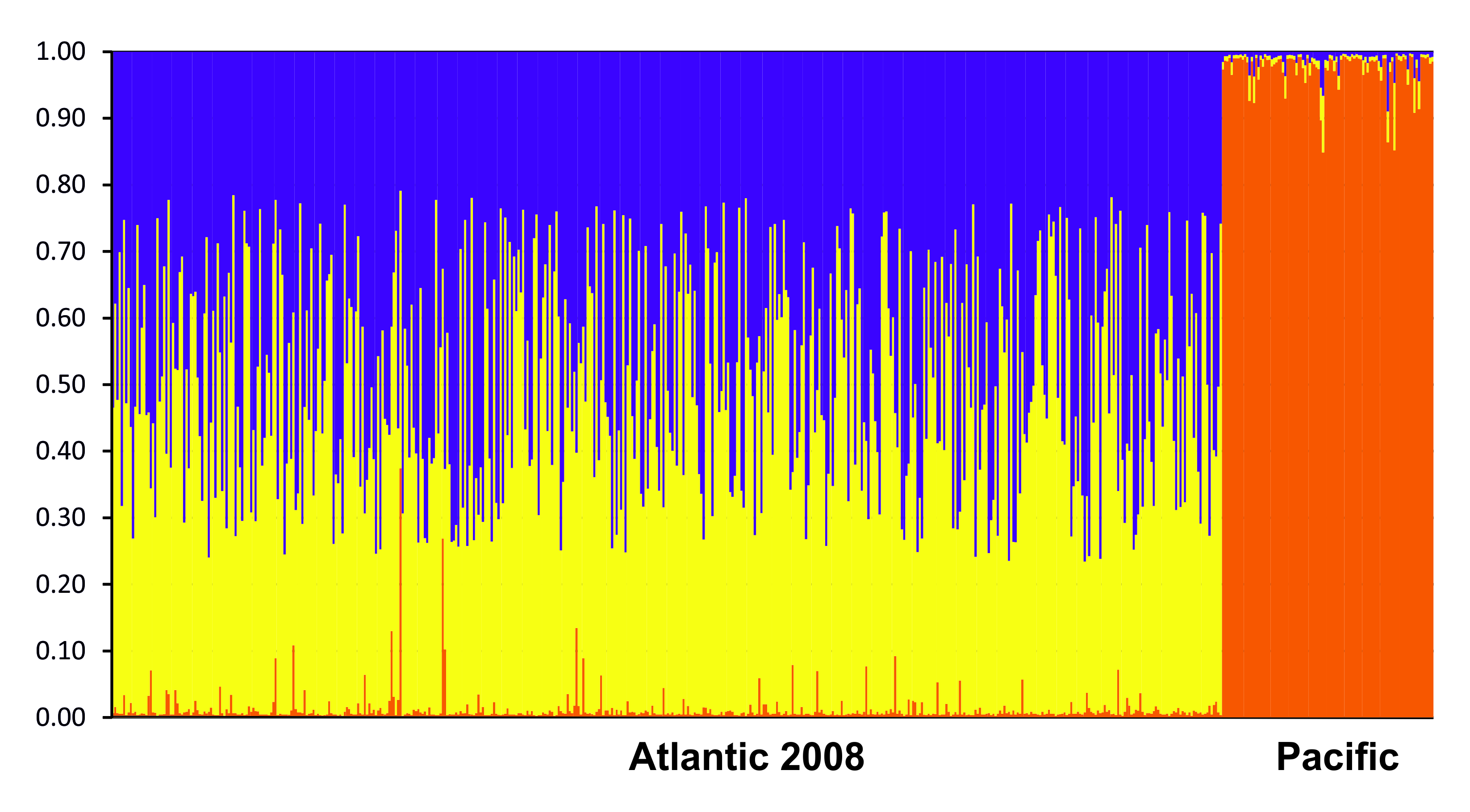 | 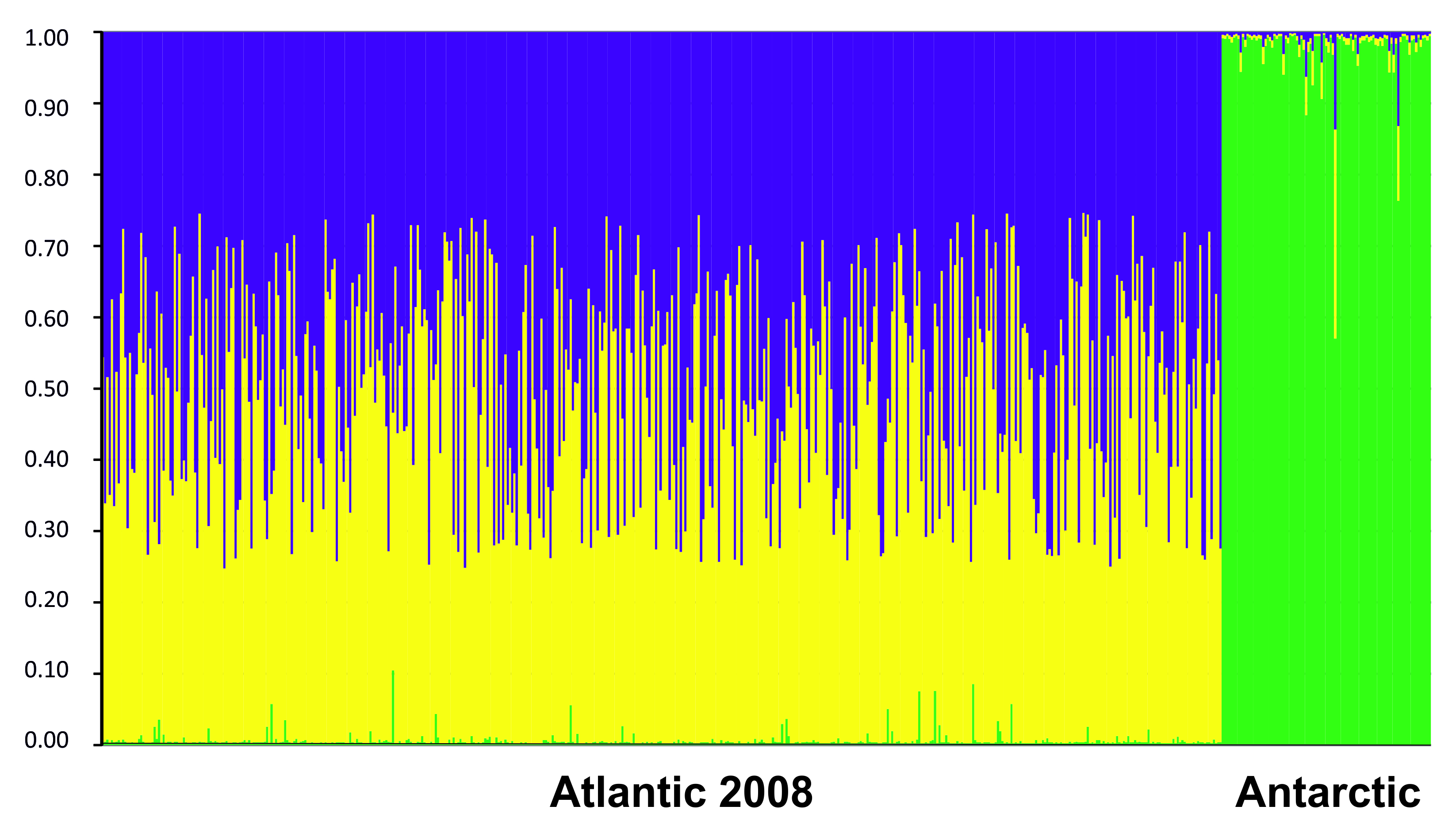 |
| 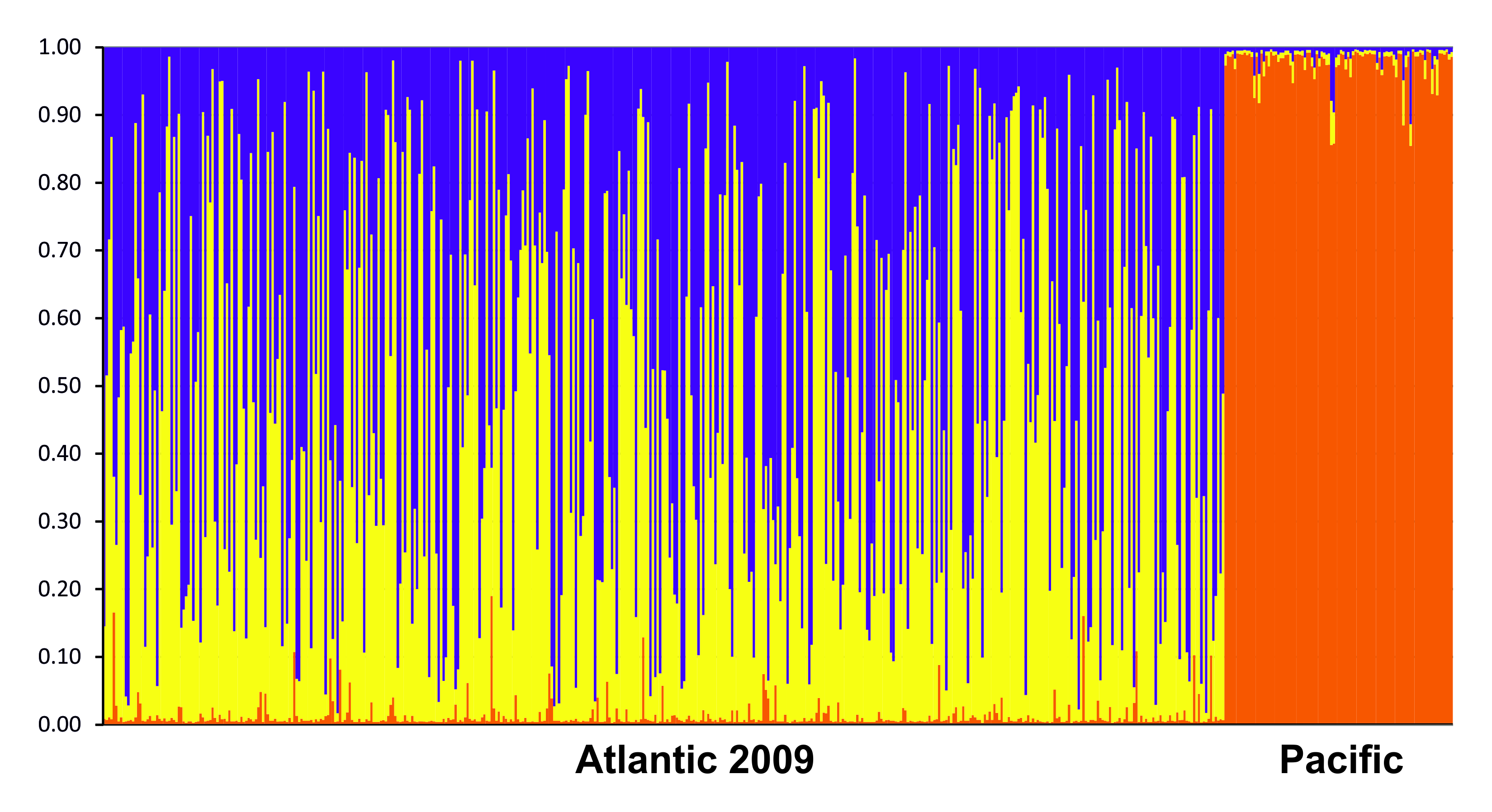 | 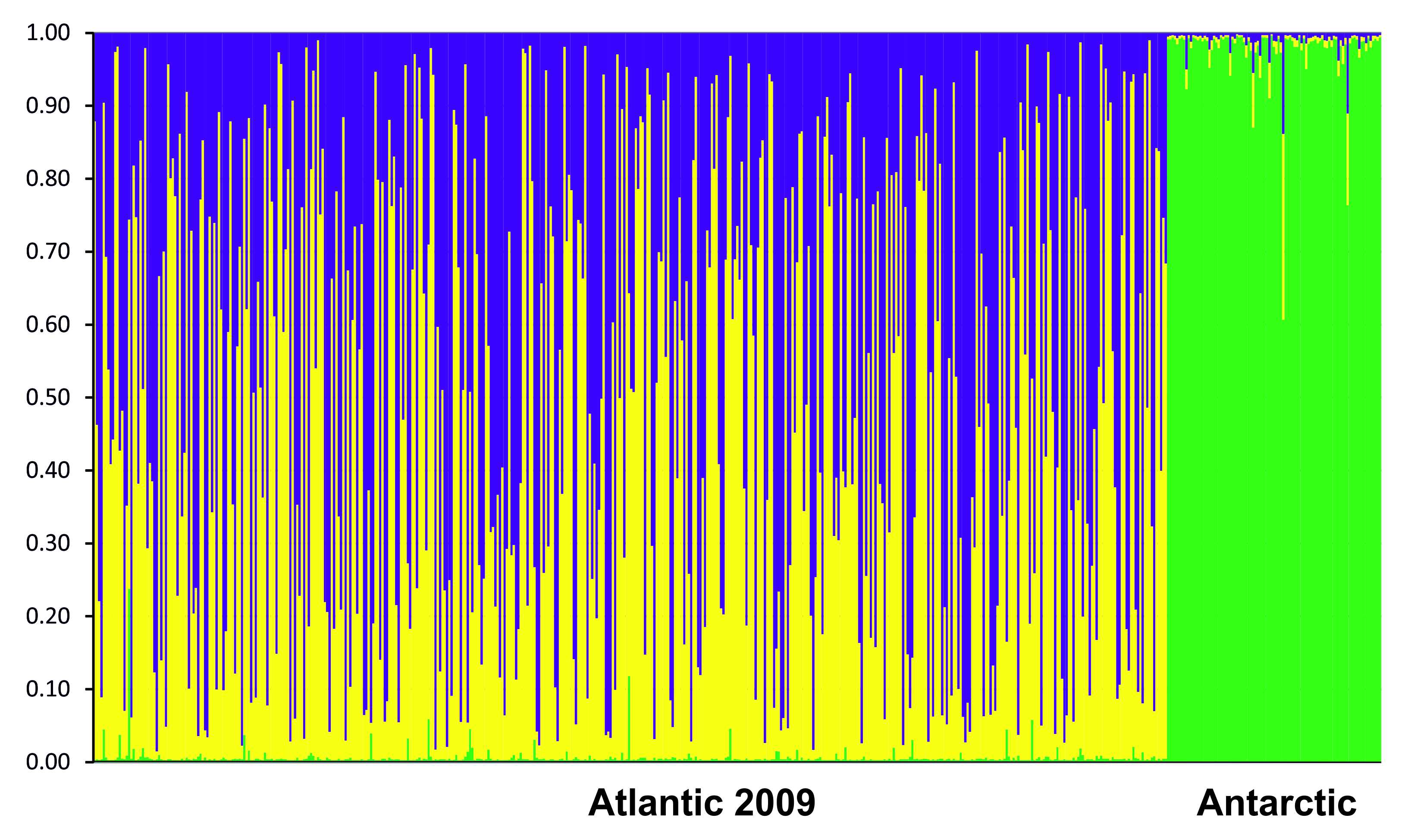 |
| 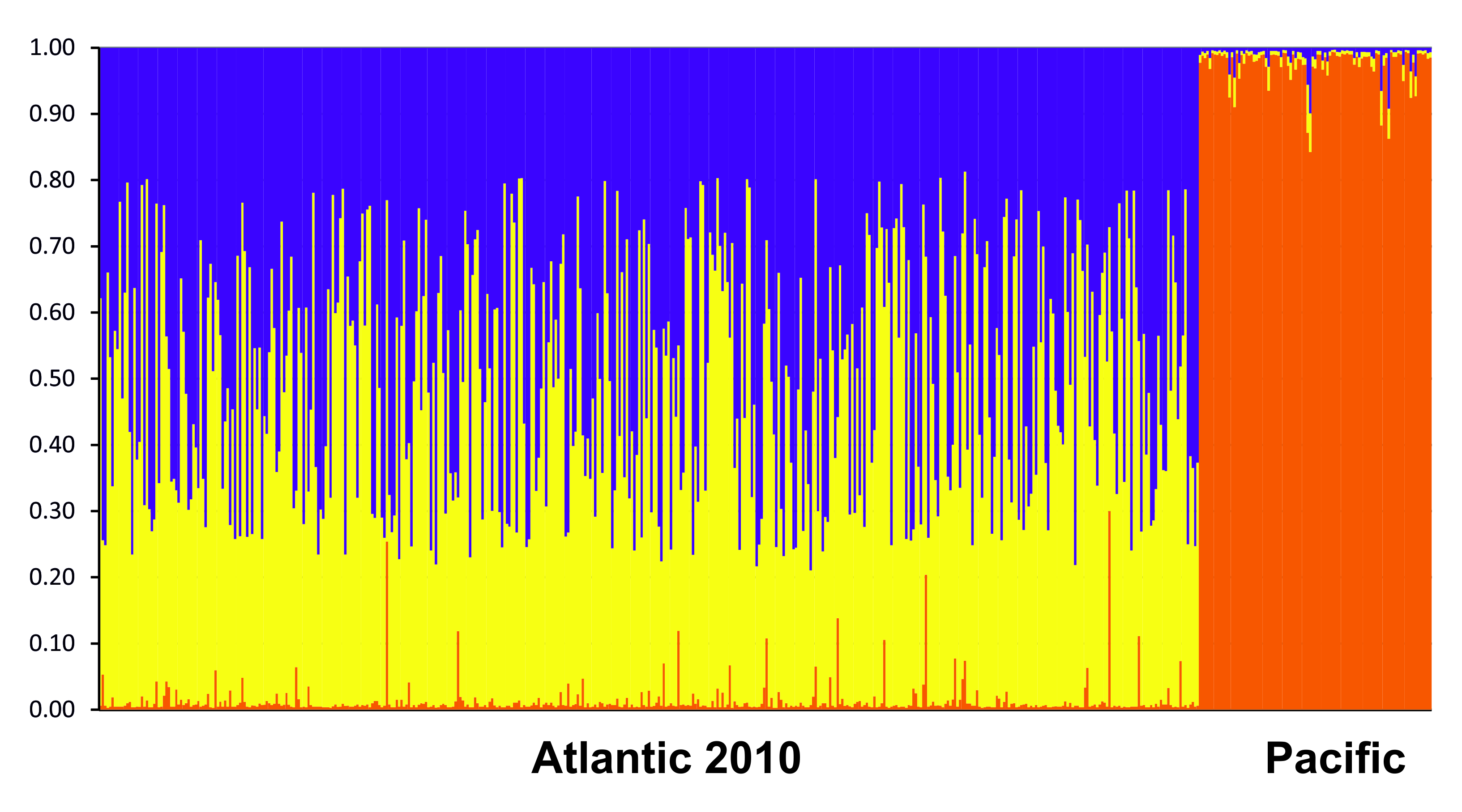 | 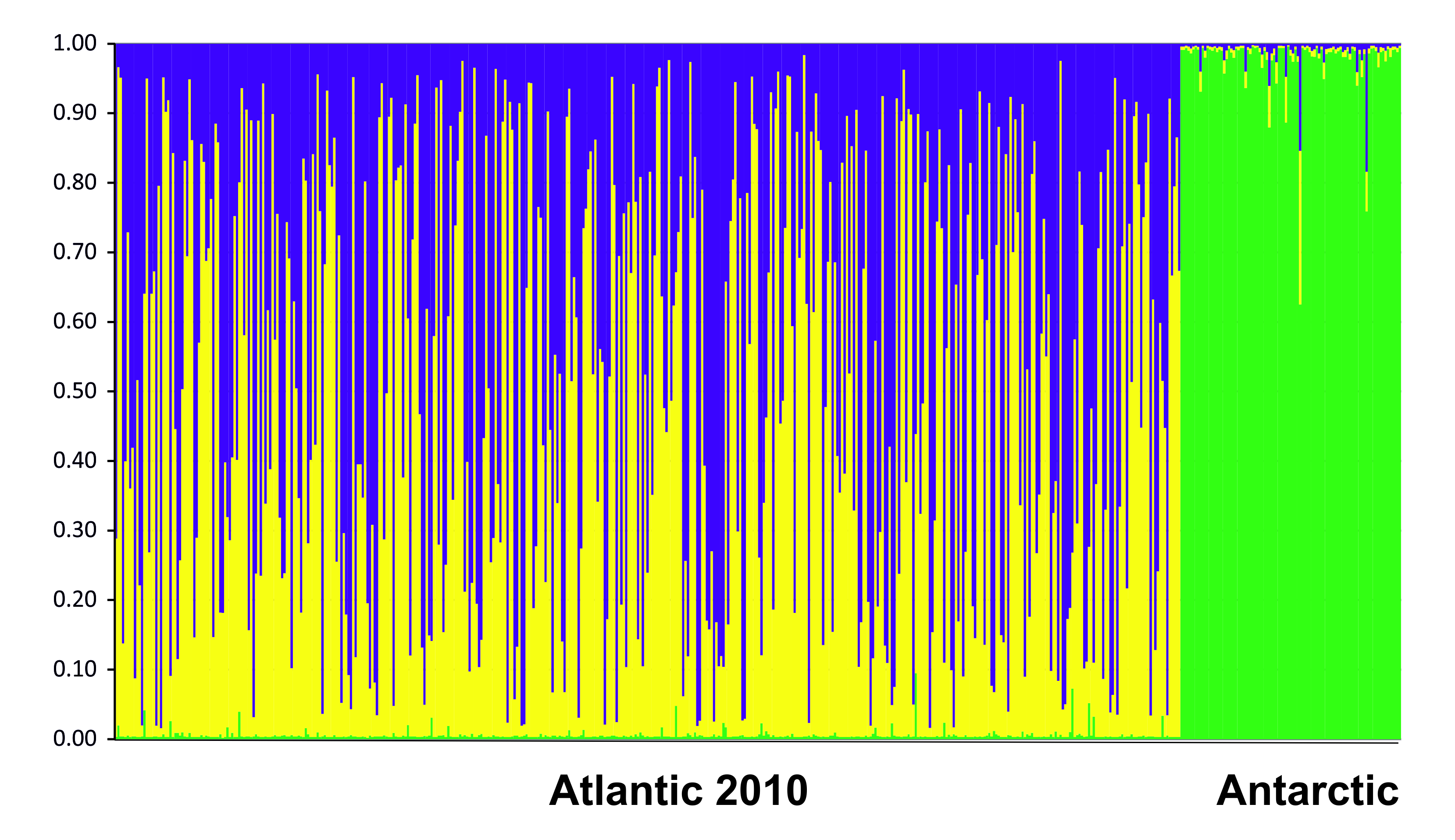 |
| 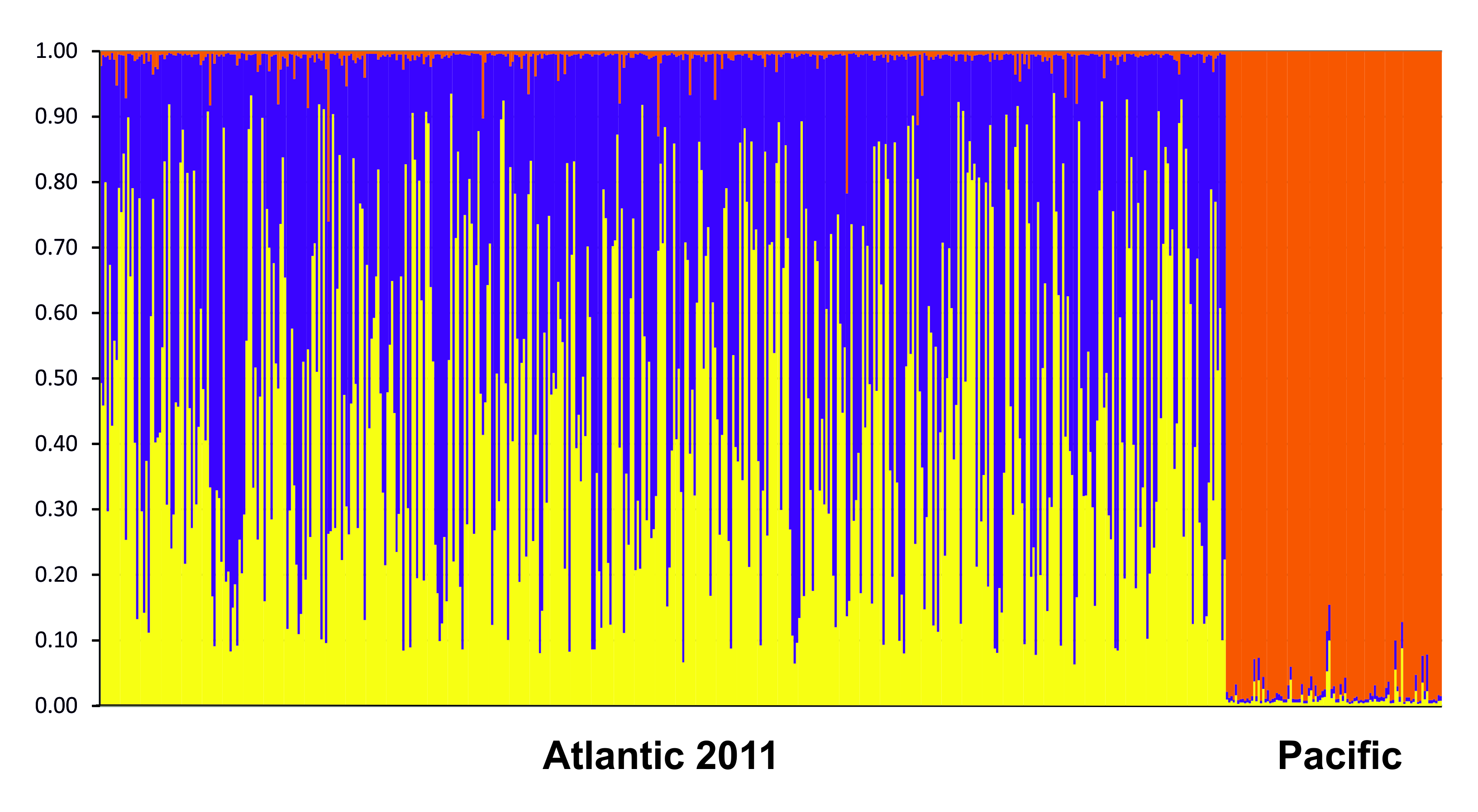 | 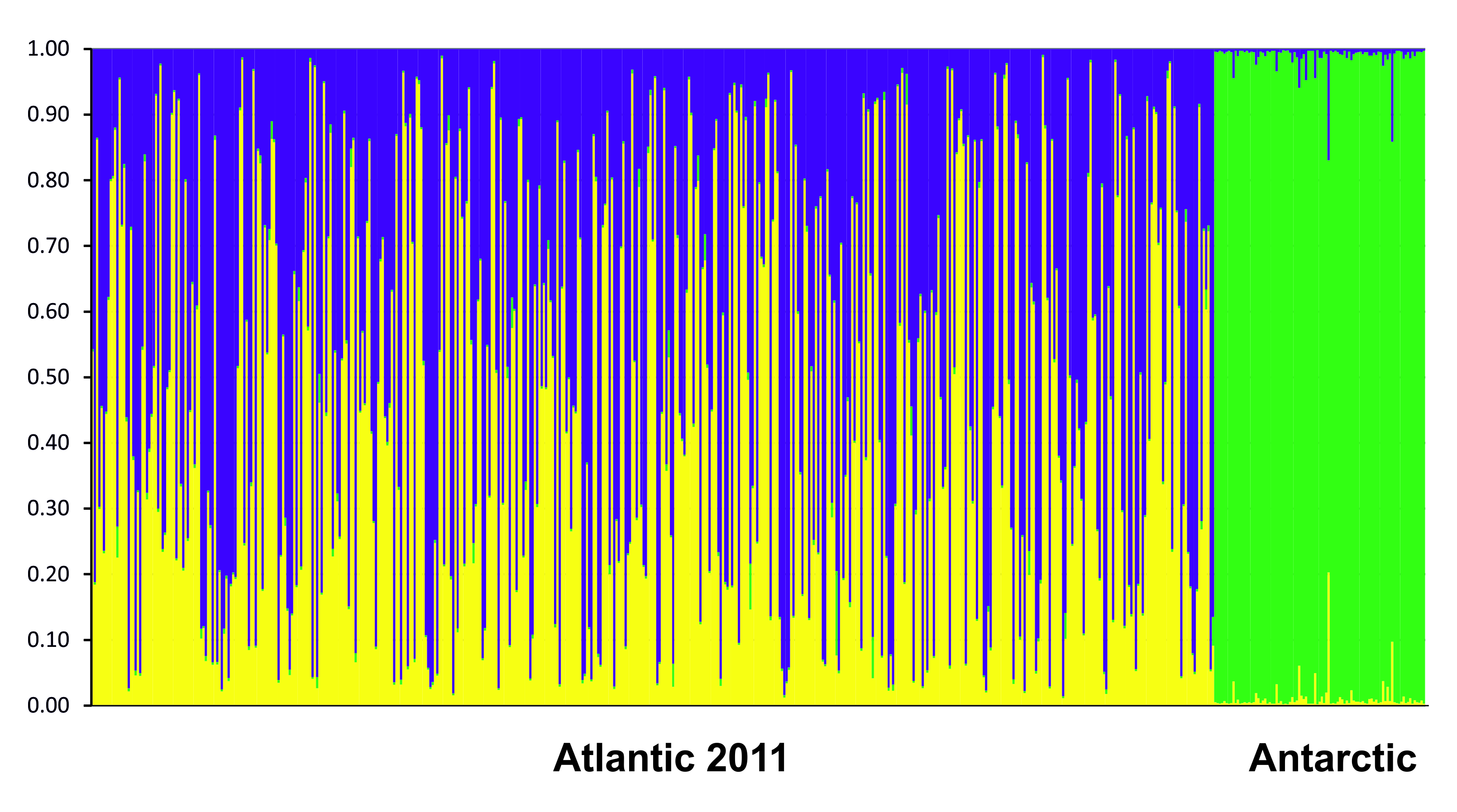 |

Fig. C. Bayesian clustering of North East Atlantic minke whale with outgroups in each year class. In the column to the left, the outgroup are 95 individuals of the subspecies Pacific minke whale (*B. a. scammoni*) whereas in the column to the right, the outgroup are 93 individuals of the Antarctic minke whale (*B. bonaerensis*). The number of clusters that best fitted the data was distinctively K=2 after Evanno’s [[5](#_ENREF_5)] test in each case.

|  | BAPS | STRUCTURE | | |
| --- | --- | --- | --- | --- |
| Area |  | no outgroup (10 microsatellites) | no outgroup (8 microsatellites) | outgroup (consensus) |
| CM |  |  |  |  |
| EB |  |  |  |  |
| EN |  |  |  |  |
| ES |  |  |  |  |
| EW |  |  |  |  |

Fig. D. Geographic distribution of individuals after different clustering methods: BAPS and STRUCTURE for microsatellites. Pie charts represent the percentage of individuals belonging to clusters 1 (dark grey) and 2 (light grey) per Management Area taking year class 2008 as an example (the full data for all the year classes is available in Table K).

|  |  |
| --- | --- |
|  |  |
|  |  |
|  |  |
|  |  |

**Fig. E.** Bayesian clustering of individuals of ten of the simulated panmictic populations that showed K=2 after Evanno’s test. Inferred ancestry of individuals was calculated after averaging ten STRUCTURE runs with CLUMPP.

| **Year 2004**  **** | **Year 2007**  **** |
| --- | --- |
| **Year 2008**  **** | **Year 2009**  **** |
| **Year 2010**  **** | **Year 2011**  **** |

Fig. F. Distribution of pairwise F_ST_ after 10000 random clustering of North Atlantic minke whale individuals per year class into two groups.

**
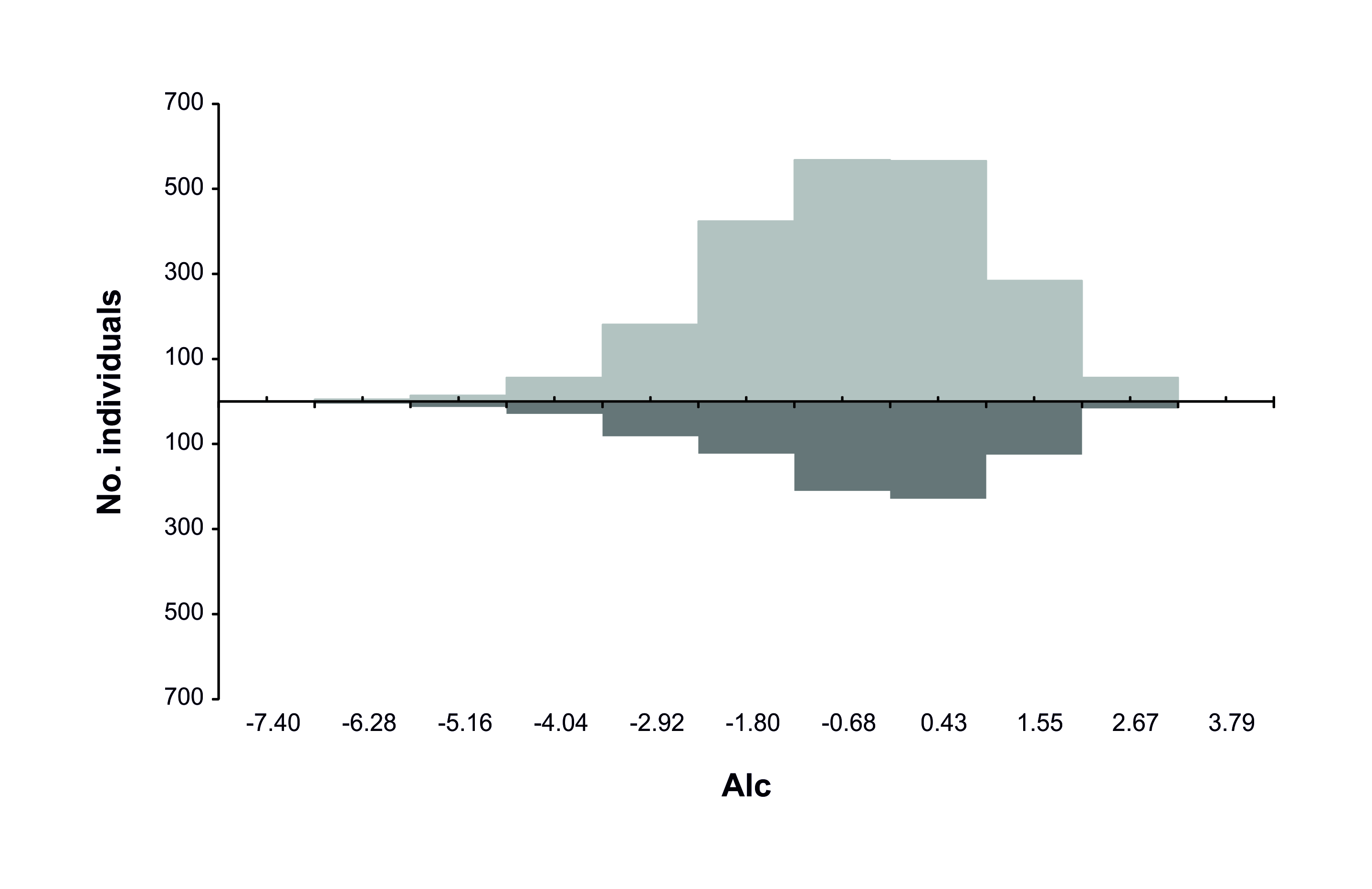
**

**Fig. G.** Frequency distributions of the corrected assignment index (AIc) for 2156 females (light grey bars above axis) and 834 males (dark grey bars below axis). AIc values differed among sexes, males having on average negative values (-0.051) and higher variance (2.90) and females positive values (0.020) with lower variance (2.35). However, Mann–Whitney U-test proved sex-biased dispersal to be non-significant (*P*>0.5).

TABLES

**Table A.** Summary result of STRUCTURE without outgroups:

1. Data set of 8 microsatellites:

|  | **LnP(D)** | | | | |  |  |
| --- | --- | --- | --- | --- | --- | --- | --- |
| **Year class** | **K=1** | **K=2** | **K=3** | **K=4** | **K=5** | **Evanno (K)** | **Evanno ΔK** |
| **2004** | -15138.10 | -15281.85 | -15537.46 | -15697.98 | -15892.51 | 2 | 5.8 |
| **2007** | -16660.00 | -16731.47 | -16901.24 | -17063.31 | -17229.91 | 2 | 8.3 |
| **2008** | -14801.76 | -15007.33 | -15279.97 | -15380.12 | -15292.04 | 3 | 3.0 |
| **2009** | -13795.81 | -13843.77 | -13931.92 | -14043.17 | -14113.58 | 2 | 8.7 |
| **2010** | -13250.30 | -13424.98 | -13514.02 | -13672.18 | -13914.68 | 2 | 3.6 |
| **2011** | -13943.03 | -14658.33 | -14740.63 | -14908.94 | -15019.38 | 2 | 16.7 |

1. Data set of 10 microsatellites:

|  | **LnP(D)** | | | | |  |  |
| --- | --- | --- | --- | --- | --- | --- | --- |
| **Year class** | **K=1** | **K=2** | **K=3** | **K=4** | **K=5** | **Evanno (K)** | **Evanno ΔK** |
| **2004** | -18152.70 | -18291.34 | -18538.75 | -18623.36 | -18875.55 | 2 | 7.6 |
| **2007** | -19877.52 | -19951.48 | -20098.10 | -20247.38 | -20381.06 | 2 | 5.7 |
| **2008** | -17627.81 | -17751.70 | -17987.74 | -18187.36 | -18277.20 | 2 | 4.5 |
| **2009** | -16492.76 | -16560.19 | -16642.55 | -16758.00 | -16864.52 | 2 | 3.7 |
| **2010** | -15832.93 | -15925.94 | -16018.93 | -16207.28 | -16386.78 | 3 | 4.1 |
| **2011** | -17352.29 | -17427.70 | -17585.63 | -17787.84 | -17961.73 | 2 | 3.7 |

**Table B.** STRUCTURE without outgroups: Clustering of individuals per year class after Evanno´s test (the two cases that showed the highest Evanno´s ∆K at K=3 are depicted in italics and analysed for K=2 for comparison): Number of individuals per cluster and range of inferred membership to each of them (in brackets). Summary of the results of the AMOVA (F_ST_ and *P*-value) conducted with Arlequin with 10000 permutations. Analyses were performed for the same sets of individuals genotyped at mtDNA. Statistically significant values were highlighted in boldface type. Negative F_ST_ values found at mtDNA were transformed into 0.

1. Data set of 8 microsatellites

|  | **No individuals** | | | **Microsatellites** | | **mtDNA** | |
| --- | --- | --- | --- | --- | --- | --- | --- |
| **Year** | **Cluster I** | **Cluster II** | **Non- assign** | **F_ST_** | **R_ST_** | **F_ST_ (Haplotype frequency)** | **F_ST_ (Tamura-Nei)** |
| 2004 | 235 | 227 | 53 | **0.0164** (<0.0001) | **0.0135** (<0.0001) | 0.0000 (0.5097) | 0.0024 (0.2882) |
|  | (0.51-0.62) | (0.51-0.63) | (0.50-0.50) |  |  |  |  |
| 2007 | 265 | 226 | 76 | **0.0121** (<0.0001) | **0.0323** (<0.0001) | 0.0000 (0.4719) | 0.0000 (0.7355) |
|  | (0.51-0.57) | (0.51-0.57) | (0.50-0.50) |  |  |  |  |
| *2008* | *215* | *190* | *93* | ***0.0135*** *(<0.0001)* | ***0.0037*** *(0.0425)* | *0.0000 (0.5315)* | *NC ^NOTE^* |
|  | *(0.51-0.55)* | *(0.51-0.56)* | *(0.50-0.50)* |  |  |  |  |
| 2009 | 212 | 168 | 86 | **0.0111** (<0.0001) | **0.0202** (0.0002) | 0.0027 (0.1143) | 0.0000 (0.6273) |
|  | (0.51-0.55) | (0.51-0.58) | (0.50-0.50) |  |  |  |  |
| 2010 | 219 | 191 | 39 | **0.0162** (<0.0001) | **0.0152** (0.0003) | 0.0038 (0.0510) | NC ^NOTE^ |
|  | (0.51-0.60) | (0.51-0.60) | (0.50-0.50) |  |  |  |  |
| 2011 | 225 | 186 | 84 | **0.0128** (<0.0001) | **0.0055** (0.0140) | 0.0000 (0.9991) | NC ^NOTE^ |
|  | (0.51-0.55) | (0.51-0.56) | (0.50-0.50) |  |  |  |  |

^NOTE^ NC (not calculated): Nucleotide composition too unbalanced for Tamura-Nei correction.

1. Data set of 10 microsatellites

|  | **No individuals** | | | **Microsatellites** | | **mtDNA** | |
| --- | --- | --- | --- | --- | --- | --- | --- |
| **Year** | **Cluster I** | **Cluster II** | **Non- assign** | **F_ST_** | **R_ST_** | **F_ST_ (Haplotype frequency)** | **F_ST_ (Tamura-Nei)** |
| 2004 | 247 | 220 | 48 | **0.0177** (<0.0001) | **0.0119** (0.0004) | 0.0000 (0.6829) | 0.0006 (0.4083) |
|  | (0.51-0.63) | (0.51-0.62) | (0.50-0.50) |  |  |  |  |
| 2007 | 263 | 228 | 76 | **0.0139** (<0.0001) | **0.0401** (<0.0001) | 0.0000 (0.8038) | 0.0006 (0.4083) |
|  | (0.51-0.58) | (0.51-0.60) | (0.50-0.50) |  |  |  |  |
| 2008 | 232 | 210 | 56 | **0.0170** (<0.0001) | **0.0073** (0.0055) | 0.0000 (0.4702) | NC ^NOTE^ |
|  | (0.51-0.59) | (0.51-0.63) | (0.50-0.50) |  |  |  |  |
| 2009 | 213 | 175 | 78 | **0.0121** (<0.0001) | **0.01226** (0.0008) | 0.0000 (1.0000) | 0.0000 (1.0000) |
|  | (0.51-0.57) | (0.51-0.59) | (0.50-0.50) |  |  |  |  |
| *2010* | *212* | *186* | *51* | ***0.0133*** *(<0.0001)* | ***0.0176*** *(<0.0001)* | *0.0016 (0.1849)* | *NC ^NOTE^* |
|  | *(0.51-0.58)* | *(0.51-0.58)* | *(0.50-0.50)* |  |  |  |  |
| 2011 | 222 | 182 | 91 | **0.0110** (<0.0001) | **0.0055** (0.0213) | 0.0000 (0.9704) | 0.0000 (0.7907) |
|  | (0.51-0.55) | (0.51-0.57) | (0.50-0.50) |  |  |  |  |

^NOTE^ NC (not calculated): Nucleotide composition too unbalanced for Tamura-Nei correction.

**Table C.** Summary statistics after STRUCTURE clustering showing total number of alleles, number of private alleles, observed heterozygosity (average ± SE), unbiased expected heterozygosity (average ± SE), and inbreeding coefficient (F_IS_) (average ± SD). We show in italics the distribution of the individuals for K=2 for the two year classes that showed the highest Evanno´s ∆K at K=3.

1. Data set of 8 microsatellites

|  | **No alleles** | | **No private alleles** | | **Ho** | | **uHe** | | **F_IS_** | |
| --- | --- | --- | --- | --- | --- | --- | --- | --- | --- | --- |
| **Year** | **CI** | **CII** | **CI** | **CII** | **CI** | **CII** | **CI** | **CII** | **CI** | **CII** |
| 2004 | 102 | 95 | 2 | 22 | 0.732 ± 0.025 | 0.801 ± 0.022 | 0.732 ± 0.023 | 0.804 ± 0.019 | 0.033 ± 0.022 | 0.003 ± -0.001 |
| 2007 | 95 | 117 | 3 | 25 | 0.745 ± 0.021 | 0.804 ± 0.026 | 0.734 ± 0.021 | 0.804 ± 0.022 | -0.017 ± 0.038 | 0.000 ± 0.023 |
| *2008* | *99* | *115* | *6* | *22* | *0.764 ± 0.027* | *0.815 ± 0.020* | *0.743 ± 0.025* | *0.798 ± 0.020* | *-0.029 ± 0.017* | *-0.023 ± 0.037* |
| 2009 | 97 | 117 | 4 | 24 | 0.751 ± 0.023 | 0.811 ± 0.027 | 0.740 ± 0.023 | 0.806 ± 0.022 | -0.017 ± 0.034 | -0.007 ± 0.035 |
| 2010 | 101 | 112 | 4 | 15 | 0.732 ± 0.024 | 0.800 ± 0.022 | 0.747 ± 0.024 | 0.790 ± 0.022 | 0.018 ± 0.023 | -0.015 ± 0.033 |
| 2011 | 90 | 114 | 0 | 24 | 0.744 ± 0.021 | 0.810 ± 0.030 | 0.740 ± 0.024 | 0.800 ± 0.023 | -0.008 ± 0.030 | -0.011 ± 0.034 |

1. Data set of 10 microsatellites

|  | **No alleles** | | **No private alleles** | | **Ho** | | **uHe** | | **F_IS_** | |
| --- | --- | --- | --- | --- | --- | --- | --- | --- | --- | --- |
| **Year** | **CI** | **CII** | **CI** | **CII** | **CI** | **CII** | **CI** | **CII** | **CI** | **CII** |
| 2004 | 101 | 114 | 5 | 18 | 0.734 ± 0.026 | 0.805 ± 0.021 | 0.731 ± 0.023 | 0.807 ± 0.018 | -0.006 ± 0.010 | 0.001 ± 0.009 |
| 2007 | 96 | 118 | 2 | 24 | 0.744 ± 0.023 | 0.806 ± 0.022 | 0.728 ± 0.023 | 0.808 ± 0.018 | -0.025 ± 0.008 | 0.000 ± 0.008 |
| 2008 | 94 | 119 | 2 | 27 | 0.755 ± 0.029 | 0.818 ± 0.020 | 0.731 ± 0.027 | 0.809 ± 0.017 | -0.034 ± 0.009 | -0.014 ± 0.012 |
| 2009 | 97 | 119 | 4 | 26 | 0.749 ± 0.022 | 0.807 ± 0.019 | 0.740 ± 0.024 | 0.806 ± 0.019 | -0.016 ± 0.012 | -0.004 ± 0.005 |
| *2010* | *94* | *114* | *1* | *21* | *0.723 ± 0.023* | *0.812 ± 0.022* | *0.738 ± 0.026* | *0.804 ± 0.018* | *0.016 ± 0.008* | *-0.013 ± 0.013* |
| 2011 | 91 | 113 | 0 | 22 | 0.739 ± 0.019 | 0.812 ± 0.025 | 0.736 ± 0.024 | 0.806 ± 0.019 | -0.008 ± 0.009 | -0.009 ± 0.010 |

**Table D.** STRUCTURE with the Pacific minke whale subspecies (*B. a. scammoni*) as an outgroup. Clustering of individuals per year class and one randomly chosen simulated panmictic population after Evanno´s test and CLUMPP averaging: Number of individuals per cluster and range of inferred membership to each of them (in brackets). Summary of the results of the AMOVA (F_ST_ and *P*-value) conducted with Arlequin with 10000 permutations. Analyses were performed for the same sets of individuals genotyped at mtDNA. Statistically significant values were highlighted in boldface type. Negative F_ST_ values found at mtDNA were transformed into 0.

|  | **No individuals** | | | **Microsatellites** | | **mtDNA** | |
| --- | --- | --- | --- | --- | --- | --- | --- |
| **Sample** | **Cluster I** | **Cluster II** | **Non- assign** | **F_ST_** | **R_ST_** | **F_ST_ (Haplotype frequency)** | **F_ST_ (Tamura-Nei)** |
| 2004 | 254 | 237 | 24 | **0.0164** (<0.0001) | **0.0127** (0.0001) | 0.0000 (0.4139) | 0.0011 (0.3654) |
|  | (0.51-0.82) | (0.51-0.82) | (0.23-0.50) |  |  |  |  |
| 2007 | 284 | 264 | 19 | **0.0111** (<0.0001) | 0.0014 (0.1262) | 0.0015 (0.1313) | 0.0000 (0.4205) |
|  | (0.51-0.97) | (0.51-0.97) | (0.17-0.50) |  |  |  |  |
| 2008 | 250 | 222 | 26 | **0.0100** (<0.0001) | **0.0329** (<0.0001) | 0.0000 (0.4291) | 0.0000 (0.6870) |
|  | (0.51-0.77) | (0.51-0.78) | (0.21-0.50) |  |  |  |  |
| 2009 | 231 | 228 | 7 | **0.0132** (<0.0001) | **0.0176** (<0.0001) | 0.0000 (0.4190) | NC |
|  | (0.51-0.98) | (0.51-0.98) | (0.38-0.50) |  |  |  |  |
| 2010 | 220 | 210 | 12 | **0.0185** (<0.0001) | **0.0155** (0.0001) | 0.0008 (0.2659) | 0.0391 (0.1061) |
|  | (0.51-0.79) | (0.51-0.80) | (0.39-0.50) |  |  |  |  |
| 2011 | 257 | 226 | 12 | **0.0130** (<0.0001) | **0.0091** (0.00099) | 0.0000 (0.4903) | 0.0000 (0.5723) |
|  | (0.51-0.93) | (0.51-0.94) | (0.26-0.50) |  |  |  |  |
| SIMPOP | 285 | 267 | 15 | **0.0145** (<0.0001) | **0.0091** (0.00020) | No data | No data |
|  | (0.51-0.98) | (0.51-0.98) | (0.19-0.50) |  |  |  |  |

^NOTE^ NC (not calculated): Nucleotide composition too unbalanced for Tamura-Nei correction.

**Table E.** STRUCTURE with Antarctic minke whale species (*B. bonaerensis*) as an outgroup. Clustering of individuals per year class and one randomly chosen simulated panmictic population after Evanno´s test and CLUMPP averaging: Number of individuals per cluster and range of inferred membership to each of them (in brackets). Summary of the results of the AMOVA (F_ST_ and *P*-value) conducted with Arlequin with 10000 permutations. Analyses were performed for the same sets of individuals genotyped at mtDNA. Statistically significant values were highlighted in boldface type. Negative F_ST_ values found at mtDNA were transformed into 0.

|  | **No individuals** | | | **Microsatellites** | | **mtDNA** | |
| --- | --- | --- | --- | --- | --- | --- | --- |
| **Sample** | **Cluster I** | **Cluster II** | **Non- assign** | **F_ST_** | **R_ST_** | **F_ST_ (Haplotype frequency)** | **F_ST_ (Tamura-Nei)** |
| 2004 | 255 | 253 | 7 | **0.0142** (<0.0001) | **0.0122** (<0.0001) | 0.0000 (0.7086) | 0.0026 (0.2607) |
|  | (0.51-0.99) | (0.51-0.97) | (0.49-0.50) |  |  |  |  |
| 2007 | 290 | 266 | 11 | **0.0101** (<0.0001) | 0.0002 (0.3281) | 0.0019 (0.1033) | 0.0040 (0.0645) |
|  | (0.51-0.98) | (0.51-0.99) | (0.49-0.50) |  |  |  |  |
| 2008 | 260 | 223 | 15 | **0.0089** (<0.0001) | **0.0422** (<0.0001) | 0.0000 (0.6062) | 0.0004 (0.4303) |
|  | (0.51-0.74) | (0.51-0.75) | (0.47-0.50) |  |  |  |  |
| 2009 | 231 | 228 | 7 | **0.0119** (<0.0001) | **0.0150** (<0.0001) | 0.0000 (0.6069) | 0.0004 (0.4346) |
|  | (0.51-0.99) | (0.51-0.99) | (0.47-0.50) |  |  |  |  |
| 2010 | 231 | 213 | 5 | **0.0156** (<0.0001) | **0.0139** (0.00020) | 0.0003 (0.3448) | NC |
|  | (0.51-0.98) | (0.51-0.98) | (0.48-0.50) |  |  |  |  |
| 2011 | 252 | 234 | 9 | **0.0125** (<0.0001) | **0.0106** (0.00059) | 0.0005 (0.2972) | NC |
|  | (0.51-0.99) | (0.51-0.99) | (0.46-0.50) |  |  |  |  |
| SIMPOP | 292 | 270 | 5 | **0.0132** (<0.0001) | **0.0102** (0.00020) | No data | No data |
|  | (0.51-0.96) | (0.51-0.96) | (0.43-0.50) |  |  |  |  |

^NOTE^ NC (not calculated): Nucleotide composition too unbalanced for Tamura-Nei correction.

**Table F.** STRUCTURE consensus clustering of individuals (*i.e.* agreement between Antarctic and Pacific outgroup clustering) into two groups per year class. Summary of the results of the AMOVA (F_ST_ and *P*-value) conducted with Arlequin with 10000 permutations. Analyses were performed for the same sets of individuals at mtDNA. Statistically significant values are highlighted in boldface type.

|  | **No individuals** | | | **Microsatelites** | | **mtDNA** | |
| --- | --- | --- | --- | --- | --- | --- | --- |
| **Year** | **Cluster I** | **Cluster II** | **Non- assign** | **F_ST_** | **R_ST_** | **F_ST_ (Haplotype frequency)** | **F_ST_ (Tamura-Nei)** |
| 2004 | 246 | 234 | 35 | **0.0163** (<0.0001) | **0.0137** (0.0001) | 0.0000 (0.5520) | 0.0034 (0.2400) |
| 2007 | 270 | 244 | 53 | **0.0119** (<0.0001) | 0.0011 (0.1543) | 0.0020 (0.1053) | 0.0020 (0.1759) |
| 2008 | 242 | 215 | 41 | **0.0102** (<0.0001) | **0.0382** (<0.0001) | 0.0000 (0.6680) | 0.0000 (0.5745) |
| 2009 | 226 | 224 | 16 | **0.0131** (<0.0001) | **0.0164** (0.0001) | 0.0000 (0.5373) | 0.0000 (0.7361) |
| 2010 | 217 | 203 | 29 | **0.0181** (<0.0001) | **0.0156** (<0.0001) | 0.0004 (0.3313) | 0.0170 (0.2510) |
| 2011 | 246 | 219 | 30 | **0.0139** (<0.0001) | **0.0102** (0.0009) | 0.0000 (0.4139) | 0.0017 (0.3188) |

**Table G.** Summary statistics after STRUCTURE consensus clustering (*i.e.* consensus between Antarctic and Pacific outgroup clustering) showing total number of alleles, allelic richness (minimum sample size), number of private alleles, observed heterozygosity (average ± SE), unbiased expected heterozygosity (average ± SE), and inbreeding coefficient (F_IS_) (average ± SD).

|  | **No alleles** | | **Ar** | | **No private alleles** | | **Ho** | | **uHe** | | **F_IS_** | |
| --- | --- | --- | --- | --- | --- | --- | --- | --- | --- | --- | --- | --- |
| **Year** | **CI** | **CII** | **CI** | **CII** | **CI** | **CII** | **CI** | **CII** | **CI** | **CII** | **CI** | **CII** |
| 2004 | 95 | 102 | 9.43 (234) | 10.66 (234) | 13 | 20 | 0.753 ± 0.023 | 0.789 ± 0.021 | 0.755 ± 0.023 | 0.780 ± 0.016 | 0.0015 ± 0.0306 | -0.0120 ± 0.0322 |
| 2007 | 94 | 106 | 9.30 (244) | 10.78 (244) | 13 | 25 | 0.781 ± 0.023 | 0.760 ± 0.024 | 0.768 ± 0.019 | 0.765 ± 0.022 | -0.0175 ± 0.0323 | 0.0051 ± 0.0368 |
| 2008 | 105 | 105 | 10.38 (215) | 10.94 (215) | 16 | 16 | 0.790 ± 0.017 | 0.781 ± 0.031 | 0.776 ± 0.020 | 0.764 ± 0.028 | -0.0216 ± 0.0339 | -0.0229 ± 0.0344 |
| 2009 | 105 | 101 | 11.03 (224) | 10.49 (224) | 20 | 16 | 0.779 ± 0.026 | 0.773 ± 0.020 | 0.768 ± 0.023 | 0.769 ± 0.021 | -0.0149 ± 0.0278 | -0.0082 ± 0.0366 |
| 2010 | 94 | 99 | 9.33 (203) | 10.55 (203) | 14 | 19 | 0.768 ± 0.032 | 0.755 ± 0.020 | 0.760 ± 0.032 | 0.770 ± 0.019 | -0.0128 ± 0.0340 | 0.0184 ± 0.0400 |
| 2011 | 100 | 98 | 10.54 (219) | 9.90 (219) | 16 | 14 | 0.780 ± 0.024 | 0.778 ± 0.021 | 0.768 ± 0.023 | 0.765 ± 0.019 | -0.0168 ± 0.0279 | -0.0179 ± 0.0217 |

**Table H.** BAPS clustering of individuals genotyped with microsatellites into two groups per year class. Summary of the results of the AMOVA (F_ST_ and *P*-value) conducted with ARLEQUIN with 10000 permutations. Analyses were performed for the same sets of individuals at mtDNA. Statistically significant values were highlighted in boldface type.

|  | **No individuals** | | **Microsatellites** | | **mtDNA** | |
| --- | --- | --- | --- | --- | --- | --- |
| **Year** | **Cluster 1** | **Cluster 2** | **F_ST_** | **R_ST_** | **F_ST_ (Haplotype frequency)** | **F_ST_ (Tamura-Nei)** |
| 2004 | 312 | 203 | **0.0059** (<0.0001) | **0.0093** (0.0014) | 0.0008 (0.2335) | 0.0004 (0.4135) |
| 2007 | 326 | 241 | **0.0090** (<0.0001) | **0.0077** (0.0012) | 0.0000 (0.4486) | **0.0082** (0.0105) |
| 2008 | 264 | 234 | **0.0097** (<0.0001) | **0.0223** (<0.0001) | **0.0060** (0.0089) | 0.0866 (0.9996) |
| 2009 | 256 | 210 | **0.0128** (<0.0001) | **0.0178** (<0.0001) | 0.0000 (0.5327) | 0.0000 (0.8373) |
| 2010 | 265 | 184 | **0.0151** (<0.0001) | **0.0033** (0.0491) | 0.0001 (0.3893) | 0.0000 (0.8295) |
| 2011 | 255 | 240 | **0.0143** (<0.0001) | **0.0043** (0.0216) | 0.0000 (0.4635) | 0.0001 ( 0.4382) |

**Table I.** Summary statistics after BAPS clustering showing total number of alleles, allelic richness (minimium sample size), number of private alleles, observed heterozygosity (average ± SE), unbiased expected heterozygosity (average ± SE), and inbreeding coefficient (F_IS_) (average ± SD).

|  | **No alleles** | | **Ar** | | **No private alleles** | | **Ho** | | **uHe** | | **F_IS_** | |
| --- | --- | --- | --- | --- | --- | --- | --- | --- | --- | --- | --- | --- |
| **Year** | **C1** | **C2** | **C1** | **C2** | **C1** | **C2** | **C1** | **C2** | **C1** | **C2** | **C1** | **C2** |
| 2004 | 113 | 75 | 10.87 (203) | 10.70 (203) | 44 | 6 | 0.791 ± 0.019 | 0.737 ± 0.027 | 0.791 ± 0.018 | 0.743 ± 0.024 | -0.0011 ± 0.0327 | 0.0080 ± 0.0315 |
| 2007 | 112 | 90 | 10.90 (241) | 10.92 (241) | 30 | 8 | 0.780 ± 0.023 | 0.769 ± 0.020 | 0.775 ± 0.020 | 0.759 ± 0.019 | -0.0062 ± 0.0269 | -0.0148 ± 0.0319 |
| 2008 | 108 | 91 | 10.72 (234) | 11.15 (234) | 32 | 15 | 0.801 ± 0.024 | 0.769 ± 0.023 | 0.782 ± 0.023 | 0.759 ± 0.021 | -0.0262 ± 0.0406 | -0.0135 ± 0.0228 |
| 2009 | 105 | 98 | 10.34 (210) | 11.10 (210) | 26 | 19 | 0.774 ± 0.020 | 0.781 ± 0.027 | 0.771 ± 0.021 | 0.767 ± 0.024 | -0.0066 ± 0.0387 | -0.0197 ± 0.0335 |
| 2010 | 105 | 89 | 10.20 (184) | 10.63 (184) | 27 | 11 | 0.765 ± 0.021 | 0.765 ± 0.025 | 0.767 ± 0.022 | 0.769 ± 0.023 | 0.0002 ± 0.0251 | 0.0035 ± 0.0441 |
| 2011 | 101 | 95 | 10.78 (240) | 10.06 (240) | 16 | 13 | 0.773 ± 0.019 | 0.781 ± 0.029 | 0.767 ± 0.019 | 0.765 ± 0.025 | -0.0060 ± 0.0188 | -0.0202 ± 0.0305 |

**Table J.** GeneClass self-assignment: Percentage of individuals genotyped at microsatellites that were correctly assignment after clustering procedures.

|  | **BAPS** | **STRUCTURE no outgroup** | | **STRUCTURE consensus** |
| --- | --- | --- | --- | --- |
| **Year** |  | **8 microsatellites** | **10 microsatellites** |  |
| 2004 | 100 | 89.4 | 90.4 | 97.5 |
| 2007 | 100 | 90.2 | 89.4 | 98.6 |
| 2008 | 100 | 83.5 | 91.4 | 85.6 |
| 2009 | 100 | 89.2 | 90.7 | 98.0 |
| 2010 | 100 | 87.6 | 88.9 | 98.6 |
| 2011 | 100 | 88.3 | 88.4 | 97.0 |

**Table K**. Number of individuals genotyped at microsatellites per Management Areas after clustering with BAPS and STRUCTURE (with and without outgroup). ND=No data.

| **Management Area** | **Year** | **BAPS** | | **STRUCTURE** | | | | | |
| --- | --- | --- | --- | --- | --- | --- | --- | --- | --- |
|  |  |  |  | **No outgroup**  **(10 micros)** | | **No outgroup**  **(8 micros)** | | **With outgroup**  **(consensus)** | |
|  |  | **C1** | **C2** | **CI** | **CII** | **CI** | **CII** | **CI** | **CII** |
| CM | 2004 | 7 | 10 | 12 | 4 | 3 | 13 | 9 | 6 |
|  | 2007 | ND | ND | ND | ND | ND | ND | ND | ND |
|  | 2008 | 13 | 17 | 14 | 13 | 11 | 14 | 13 | 16 |
|  | 2009 | ND | ND | ND | ND | ND | ND | ND | ND |
|  | 2010 | 1 | 0 | ND | ND | 1 | 0 | 1 | 0 |
|  | 2011 | ND | ND | ND | ND | ND | ND | ND | ND |
| EB | 2004 | 70 | 53 | 58 | 53 | 52 | 54 | 65 | 49 |
|  | 2007 | 16 | 12 | 17 | 6 | 15 | 9 | 13 | 11 |
|  | 2008 | 12 | 8 | 10 | 8 | 12 | 7 | 12 | 6 |
|  | 2009 | 2 | 1 | 1 | 2 | 0 | 2 | 1 | 2 |
|  | 2010 | 11 | 6 | 8 | 8 | 8 | 7 | 10 | 6 |
|  | 2011 | 49 | 47 | 41 | 28 | 40 | 36 | 44 | 44 |
| EN | 2004 | 54 | 27 | 41 | 32 | 34 | 41 | 38 | 38 |
|  | 2007 | 49 | 42 | 40 | 39 | 43 | 38 | 49 | 36 |
|  | 2008 | 47 | 39 | 38 | 40 | 39 | 33 | 43 | 39 |
|  | 2009 | 30 | 19 | 18 | 21 | 21 | 19 | 23 | 24 |
|  | 2010 | 18 | 9 | 14 | 12 | 13 | 10 | 9 | 16 |
|  | 2011 | 8 | 4 | 7 | 4 | 6 | 5 | 7 | 5 |
| ES | 2004 | 64 | 45 | 54 | 44 | 46 | 50 | 55 | 47 |
|  | 2007 | 164 | 112 | 126 | 114 | 127 | 110 | 133 | 123 |
|  | 2008 | 111 | 109 | 105 | 90 | 91 | 80 | 109 | 91 |
|  | 2009 | 129 | 114 | 108 | 92 | 109 | 90 | 120 | 115 |
|  | 2010 | 149 | 115 | 121 | 113 | 123 | 114 | 128 | 119 |
|  | 2011 | 97 | 87 | 89 | 69 | 85 | 70 | 96 | 77 |
| EW | 2004 | 117 | 68 | 82 | 87 | 92 | 77 | 79 | 94 |
|  | 2007 | 97 | 75 | 80 | 69 | 80 | 69 | 75 | 74 |
|  | 2008 | 81 | 61 | 65 | 59 | 62 | 56 | 65 | 63 |
|  | 2009 | 95 | 76 | 86 | 60 | 82 | 57 | 80 | 85 |
|  | 2010 | 86 | 54 | 69 | 53 | 74 | 60 | 69 | 62 |
|  | 2011 | 101 | 102 | 85 | 81 | 94 | 75 | 99 | 93 |

**Table L.** Matrix of numbers and percentage of coincident individuals when comparing the three clustering methods: BAPS, STRUCTURE without outgroup (STR), and STRUCTURE with outgroup (STR consensus). The percentage of coincident individuals was calculated by dividing the number of by the lowest number of individuals in the corresponding cluster. STRUCTURE analyses were performed with 8 microsatellites.

| **YEAR** | **CLUSTERS** | **APPROACH** | **BAPS** | **STR** |
| --- | --- | --- | --- | --- |
| **2004** | CI | STR | 136 (67%) | **** |
|  |  | STR consensus | 121 (60%) | 158 (67%) |
|  | CII | STR | 178 (78%) | **** |
|  |  | STR consensus | 166 (71%) | 141 (62%) |
| **2007** | CI | STR | 141 (59%) | **** |
|  |  | STR consensus | 202 (75%) | 139 (52%) |
|  | CII | STR | 153 (68%) | **** |
|  |  | STR consensus | 155 (64%) | 104 (46%) |
| **2008** | CI | STR | 129 (60%) | **** |
|  |  | STR consensus | 126 (54%) | 109 (51%) |
|  | CII | STR | 128 (67%) | **** |
|  |  | STR consensus | 126 (59%) | 105 (55%) |
| **2009** | CI | STR | 125 (59%) | **** |
|  |  | STR consensus | 201 (90%) | 107 (50%) |
|  | CII | STR | 79 (47%) | **** |
|  |  | STR consensus | 177 (84%) | 84 (50%) |
| **2010** | CI | STR | 104 (57%) | **** |
|  |  | STR consensus | 142 (77%) | 131 (60%) |
|  | CII | STR | 127 (66%) | **** |
|  |  | STR consensus | 171 (84%) | 103 (54%) |
| **2011** | CI | STR | 120 (53%) | **** |
|  |  | STR consensus | 181 (83%) | 117 (53%) |
|  | CII | STR | 107 (58%) | **** |
|  |  | STR consensus | 209 (85%) | 108 (58%) |

**Table M**. STRUCTURE clustering of individuals in the ten randomly selected simulated panmictic populations showing K=2 after Evanno’s test. Number of individuals per cluster and range of inferred membership to each of them (in brackets); number of non-assigned individuals (and % of the total). Summary of the results of the AMOVA (F_ST_ and *P*-value) conducted with Arlequin with 10000 permutations. Statistically significant values were highlighted in boldface type.

| **Population** | **Highest LnP(K)** | **Evanno K** | **∆K** | **Cluster 1** | **Cluster 2** | **Non-assigned individuals** | **F_ST_** | ***P*** |
| --- | --- | --- | --- | --- | --- | --- | --- | --- |
| SimPopA | 1 | 2 | 5.5 | 260 | 232 | 75 (13%) | **0.017** | <0.0001 |
|  |  |  |  | (0.51-0.56) | (0.51-0.58) |  |  |  |
| SimPopB | 1 | 2 | 13.5 | 243 | 218 | 106 (19%) | **0.012** | <0.0001 |
|  |  |  |  | (0.51-0.55) | (0.51-0.57) |  |  |  |
| SimPopC | 1 | 2 | 7.1 | 261 | 212 | 94 (17%) | **0.015** | <0.0001 |
|  |  |  |  | (0.51-0.56) | (0.51-0.59) |  |  |  |
| SimPopD | 1 | 2 | 8 | 264 | 205 | 98 (17%) | **0.016** | <0.0001 |
|  |  |  |  | (0.51-0.56) | (0.51-0.59) |  |  |  |
| SimPopE | 1 | 2 | 2 | 263 | 249 | 55 (10%) | **0.014** | <0.0001 |
|  |  |  |  | (0.51-0.59) | (0.51-0.63) |  |  |  |
| SimPopF | 1 | 2 | 2 | 264 | 258 | 45 (8%) | **0.020** | <0.0001 |
|  |  |  |  | (0.51-0.64) | (0.51-0.63) |  |  |  |
| SimPopG | 1 | 2 | 4.8 | 254 | 248 | 65 (11%) | **0.017** | <0.0001 |
|  |  |  |  | (0.51-0.58) | (0.51-0.58) |  |  |  |
| SimPopH | 1 | 2 | 4 | 262 | 245 | 60 (11%) | **0.017** | <0.0001 |
|  |  |  |  | (0.51-0.59) | (0.51-0.61) |  |  |  |
| SimPopI | 1 | 2 | 10.7 | 270 | 256 | 41 (7%) | **0.015** | <0.0001 |
|  |  |  |  | (0.51-0.62) | (0.51-0.61) |  |  |  |
| SimPopJ | 1 | 2 | 1.9 | 243 | 232 | 92 (16%) | **0.015** | <0.0001 |
|  |  |  |  | (0.51-0.58) | (0.51-0.58) |  |  |  |
